# Spatio‐Temporal Variation in Aerial Arthropod Abundance Revealed by Weather Radars

**DOI:** 10.1111/gcb.70425

**Published:** 2025-10-27

**Authors:** Mansi Mungee, Maryna Lukach, Chris Shortall, James R. Bell, Elizabeth J. Duncan, Freya I. Addison, Lee E. Brown, William E. Kunin, Christopher Hassall, Ryan R. Neely

**Affiliations:** ^1^ School of Geography and Water@Leeds, Faculty of Environment University of Leeds Leeds UK; ^2^ Azim Premji University Bhopal Madhya Pradesh India; ^3^ National Centre for Atmospheric Science University of Leeds Leeds UK; ^4^ Royal Meteorological Institute (RMI) Uccle Belgium; ^5^ School of Earth and Environment, Faculty of Environment University of Leeds Leeds UK; ^6^ Rothamsted Insect Survey, Rothamsted Research Harpenden UK; ^7^ Centre for Applied Entomology, Parasites and Pathogens, School of Life Sciences Keele University Keele UK; ^8^ School of Biology, Faculty of Biological Sciences University of Leeds Leeds UK

**Keywords:** arthropod declines, insect migration, radar entomology, spatio‐temporal ecology, United Kingdom

## Abstract

Arthropod declines have been reported widely; however, a lack of comprehensive data has hindered our ability to assess their large‐scale generality and drivers. Here, we used a novel and freely available dataset—atmospheric scans from a network of meteorological radars—to quantify aerial abundance of both diurnal and nocturnal arthropods across the United Kingdom, spanning different geographic regions and land cover types. Based on observations between 2014 and 2021, and across more than 35,000 km^2^, we estimate numbers of arthropods flying over the UK at heights between 500 and 700 m above ground at 1.12 (±0.01) × 10^13^ individuals during the diurnal (0800–1400 UTC) and 5.02 (±0.01) × 10^12^ during the nocturnal (including dusk, 1800–2200 UTC) period, showing significant spatial heterogeneity. Although spatial patterns differed, both diurnal and nocturnal arthropods increased in the south and declined mainly in the far north; on average, only nocturnal arthropods showed an overall decline. Aerial abundance of both diurnal and nocturnal arthropods showed positive relationships with woodland, grassland, and urban land cover, and negative relationships with artificial light intensity and arable land cover. Our study highlights the importance of spatial variation in temporal biodiversity trends and illustrates the need for comparative studies between nocturnal and diurnal arthropods. Notably, by extracting vertical profiles of radar reflectivity and polarization signatures, we demonstrate how weather radar datasets can be used to quantify aerial arthropod abundance, detect diurnal and seasonal activity patterns, and examine their environmental drivers across large spatial and temporal scales.

## Graphical abstract

Alarming arthropod decline has been reported from around the world, but we lack conensus on the generality, severity, and potential causes of these declines. We use weather radars for large‐scale aerial arthropod monitoring across the United Kingdom and show that, on average, nocturnal arthropods showed a decline in abundance, while diurnal arthropods did not exhibit a significant decrease over an 8‐year period (2014–2021). Widespread spatio‐temporal variations were driven by light pollution intensity, habitat type, and climate. Radar monitoring of aerial arthropods offers exciting prospects for continental or global biodiversity monitoring in the future, facilitating a new understanding of biodiversity loss.

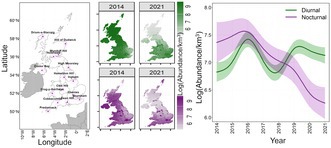

## 1 Introduction

Arthropods dominate terrestrial, freshwater and aerial environments, making up 80% of known species (Stork 2018) and almost half of global animal biomass (Bar‐On et al. 2018). There have been increasing reports of declines in arthropod (and specifically insect) populations from around the globe, but the generality of this phenomenon, including its rate, magnitude, and extent, remains poorly understood across large spatial and temporal scales (Simmons et al. 2019). Arthropods are a hyper‐abundant and hyper‐diverse group, and current monitoring methods are limited by high costs and restricted spatial and taxonomic coverage (Montgomery et al. 2020). Furthermore, the diverse metrics used to assess declines, such as species richness, occupancy, biomass, and abundance, are not directly comparable, presenting challenges to interpret and respond to the wide variability of reported trends (Didham et al. 2020). Notably, alarming trends have primarily been reported in total biomass and abundance, which are critical as they strongly impact ecosystem services (Hallmann et al. 2017). This raises severe concerns among scientists and policymakers because arthropods play crucial roles in ecosystems as pollinators, decomposers, and as a vital food source for numerous organisms higher up in the trophic web (Losey and Vaughan 2006). Enhanced understanding of drivers and consequences of arthropod declines at large scales is therefore essential for developing effective conservation strategies and mitigating potential ecological and societal disruptions

Empirical studies show that arthropods are affected by many different and interacting aspects of their environment such as climate, land cover change, invasive species, insecticides, and light pollution (Kehoe et al. 2021). However, much of our understanding about the relative effects of these drivers comes from studies either local in scale (e.g., point sampling), or utilizing presence‐only occupancy records, or by employing space‐for‐time substitution (Blüthgen et al. 2022). Few studies have simultaneously compared temporal trends in arthropod abundances across multiple habitat types and across large spatial extents (Bell et al. 2020; Uhler et al. 2021). Nonetheless, understanding these relationships is critical for conservation strategies aiming to mitigate biodiversity loss (Wagner 2020).

Radar‐based monitoring is an established tool for studying aerial animals and may provide a robust methodology for large‐scale, standardized arthropod monitoring (Bauer et al. 2017). Most recent studies have used vertical‐looking radars (VLR), which have generated considerable insights into aerial arthropod movement and abundance (Hu et al. 2016; Knop et al. 2023), but which provide limited spatial coverage. On the other hand, weather surveillance radars (WSRs), intended to monitor meteorological phenomena, use existing infrastructure without extra costs and provide unprecedented spatial coverage over thousands of square kilometers for broad‐scale biodiversity monitoring (Dokter et al. 2018). For example, the North American NEXRAD WSR network has been used to generate biologically meaningful data on bird phenology (Schools et al. 2012), migration (Schools et al. 2012; Sivakumar et al. 2021), demography (Nilsson et al. 2021), and epidemiology (McCuen et al. 2021) at national scales. With the advent of dual‐polarization capabilities, where radars transmit and receive both horizontal and vertical pulses to distinguish the elongated shapes of insects from the more spherical signatures of precipitation, WSR networks have also been used to map the emergence and migration of arthropods (Boulanger et al. 2017; Stepanian et al. 2020).

**FIGURE 1:**
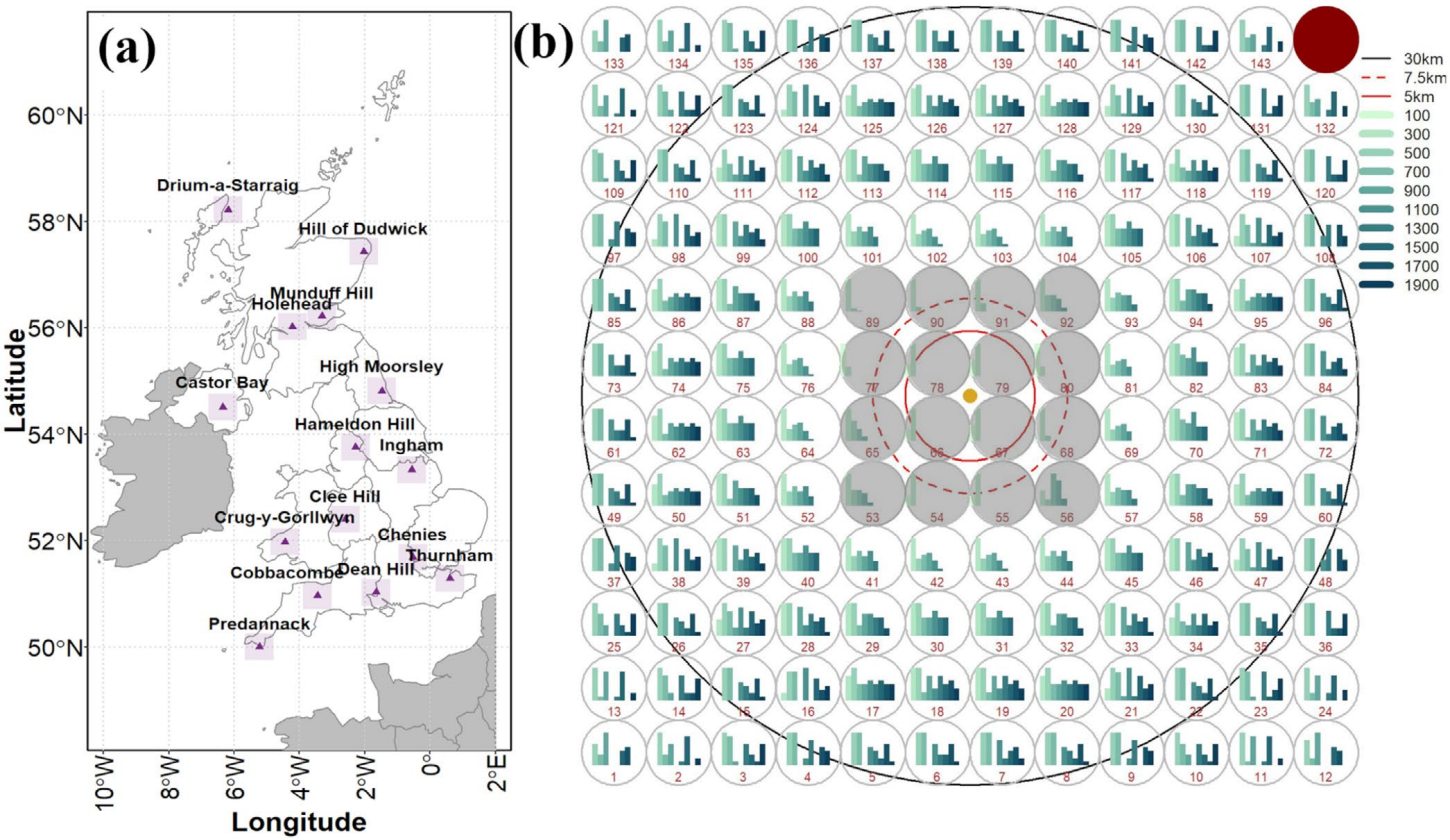
(a) Map showing locations of 15 weather radars across the UK, and (b) a 12 × 12 lattice of the different Columnar Vertical Profiles (CVPs) around the radar used for estimating aerial arthropod abundance in the present study. (a) Dual polarized data from 15 UKMO‐Radars (purple triangles) was processed from a fixed region around the radar (purple squares overlaid on the triangle, each corresponding to the region covered by a 12 × 12 CVP lattice as shown in b). (b) Around each radar, 144 Columnar Vertical Profiles (CVPs) of 5 km diameter were generated. The spatial coordinates for each CVP were obtained by creating a regular grid with the coordinates of each radar as the centroid (golden dot in the centre). The outer black circle represents the 30 km buffer where the radar beam retains sufficient resolution for stratified height analysis. The height of the bars within each CVP corresponds to the number of voxels available across different heights (see legend for heights in meters). The number of voxels vary with the range due to the beam height and broadening, hence both the height as well as the number of bars is variable across CVPs. The innermost CVPs closest to the radar (within a 5 km radius; solid red circle) were removed from all downstream analysis due to the highest likelihood of echoes from ground clutter. A further 12 CVPs falling within the 7.5 km radius (marked by the dashed red circle) were excluded from all radars due to insufficient vertical coverage of the radar beam. One CVP in the upper‐right corner (highlighted in red) could not be processed for any radar due to technical issues.

Here, we demonstrate how observations from a national network of WSRs can be used to provide robust quantitative estimates of aerial arthropod abundance across vast spatial scales and at high temporal (twice a day) frequencies. We analyzed 8 years of data (2014–2021) from 15 WSRs (Figure 1a) spanning more than 35,000 km^2^ and 10° in latitude, which represented a diverse variety of habitat types, including woodland, agricultural, and urban areas over which insects and other arthropods flew or were transported. We derived sub‐daily data describing abundance trends across the UK, making it the most comprehensive spatial investigation for both diurnal and nocturnal arthropods using a common method. The resulting datasets were used to answer three primary questions: (i) what is the abundance of aerial arthropods across the UK? (ii) have there been significant changes in abundances over the studied time period? and (iii) what are the likely spatio‐temporal drivers of any changes? We validate our analysis using long‐term, standardized monitoring of aerial arthropod abundance from a suction trap situated close to a WSR station. Our approach provides a benchmark for directing future research efforts towards the long‐term and broad‐scale investigation of overall arthropod abundance patterns using standardized, homogeneous, and openly available datasets at an unprecedented spatial scale and temporal resolution.

## 2 Materials and Methods

### 2.1 UKMO Radar Network

The UK Met Office (UKMO) operates a network of 15 weather surveillance radars, which provide complete airspace coverage over England, Wales, Scotland, and Northern Ireland (Figure 1a) (Harrison et al. 2000; Met Office 2003). Each Doppler radar is a Doppler, C‐Band (wavelength (*λ*) = 5.3 cm), dual‐polarization, monostatic radar which provides near‐continuous polarimetric measurements of differential reflectivity (Z_DR_), co‐polar correlation coefficient (ρ_HV_) and phase differential (Φ_DP_), along with the standard legacy variables of single‐polarized radars, that is, reflectivity factor (Z) and radial velocity (V). Ecological application of weather radar, especially for birds, has been the subject of several previous works (Boulanger et al. 2017; Dokter et al. 2018; Nilsson et al. 2021; Schools et al. 2012; Sivakumar et al. 2021; Stepanian et al. 2020), and, therefore, here we have only aimed to describe the unique specifications of the UKMO radars.

The raw data are disseminated in the form of plan position indicator (PPI) scans—that is, a single 360° (azimuthal) scan carried out for a fixed elevation angle and repeated over a series of different angles. The PPIs are averaged to 600 m range gates and 1° in azimuth, close to the radar beam width of 1.1°. However, for our ecological analysis, we were interested in observing the data at a fixed azimuth and over multiple elevations, that is, at a fixed location in spatial coordinates and across different heights over that location. We generated columnar vertical profiles (CVPs; described below) of all polarimetric variables using PPI scans from different elevation angles (typically between 0.5° and 4.0°) sampled on long pulse mode (pulse length = 2.0 μs; range covered = 250 km) and with a 600 m gate resolution every 5 min.

### 2.2 Columnar Vertical Profiles (CVPs)

CVPs—4D slices of data represented with latitude, longitude, time, and height—were generated following the approach of Murphy et al. (2020). Data from within the 600 m × 1° sectors were azimuthally averaged and projected to the CVP center, resulting in a vertical profile. The mean values were assigned as the profile value for different height bands, each 200 m deep (between 100 and 2100 m). Although technically speaking, columns are not circular and not strictly vertical, for simplicity and homogeneity of calculations, a circular representation is selected. Cylindrical columns can be considered as the volume representing a subset of voxels (i.e., volume pixels). We chose a column radius of 2.5 km and a vertical resolution (step‐size or height) of 200 m as the optimum trade‐off between sector size and step size, which facilitates uniform data averaging and projection (more details on CVP calculation and this selection criterion are discussed in Supporting Information: Section S1). This approach allows us to examine fine‐scale variation in polarimetric variables (to within a 2.5 km horizontally and 200 m in height) and consequently in arthropod densities. This level of detail can be valuable for identifying the environmental drivers behind the observed variations.

For each radar, we generated 144 CVPs arranged in a 12 × 12 grid within a 60 × 60 km bounding box, centered on the radar's coordinates (Figure 1b). This spatial extent was chosen because radar sensitivity declines beyond 30 km, often requiring ad‐hoc corrections that are unreliable for detecting sparse populations of small insects. Within a 30 km radius, the radar beam's vertical resolution is adequate for estimating abundance across discrete height bands (Kilambi et al. 2018). Applying this protocol across all 15 WSRs in the UKMO network yielded a total of 2160 CVPs (144 per radar). One CVP in the upper right corner (Figure 1b) could not be processed for any radar due to technical limitations, leaving 2145 CVPs for downstream processing.

As mentioned above, within a CVP, data from multiple elevation angles are azimuthally averaged and projected to the CVP center. However, due to the radar beam angle and beam broadening, the number of voxels at different heights varies with the range. We therefore removed 16 central CVPs (4 × 4 grid around the radar; Figure 1b), where few or no voxels could be surveyed at greater heights. This resulted in a loss of data but did not bias our results, as it affected the same locations across all radars, and the number of CVPs per radar remained constant. We also removed additional CVPs for which an obstruction in the radar beam would result in severe ground clutter and shadowing, which can lead to issues when extracting comparatively weak arthropod echoes. Because obstructions caused by hills are typically long‐lasting, we used a UK‐wide, 90 m Digital Terrain Model (DTM) to further remove 84 CVPs across different radars in which potential sources of obstruction were identified (Zrnic and Ryzhkov 1998; Supporting Information: Section S1). The final dataset thus consisted of (127 × 15) − 84 = 1821 CVPs in total. With the spatial area under each CVP = 19.62 km^2^ (π × 2.5^2^), this resulted in a complete spatial coverage of 35,728 km^2^ across the UK (~15% of the country's area) above which aerial arthropod abundances were estimated.

### 2.3 CVP Processing

We removed all meteorological signals that could be attributed to precipitation using the ‘DR‐Filtering’ method developed by Kilambi et al. (2018). A depolarization ratio (DR) was calculated using polarimetric variables Z_DR_ and ρ_HV_, and all data below a DR threshold of −12.5 dB were identified as precipitation and removed (Kilambi et al. 2018; Stepanian et al. 2020). We also removed all data with extremely high reflectivity factors (> 45 dBZ), which are often associated with heavy rainfall but may not be efficiently captured by the depolarization ratio (Kilambi et al. 2018; Figure S1). We used differential reflectivity (Z_DR_) to remove all birds from the resulting data. High positive values of Z_DR_ can be generally attributed to arthropods due to their somewhat more elongated body plans, with values ranging between 2 and 10 dB commonly observed (Dokter et al. 2011; Mäkinen et al. 2022; Melnikov et al. 2015; Stepanian et al. 2020; Zrnic and Ryzhkov 1998). For example, Dokter et al. (2011) used a threshold of 3 dB to filter out arthropods for studying bird migrations; for the decidedly more elongate mayflies, Stepanian et al. (2020) used a Z_DR_ threshold of 5 dB. For UK arthropods, we used a conservative threshold of 3 dB to reduce co‐occurring bird signatures.

We used seasonal and diurnal truncations to restrict our data to periods of known high arthropod activity across the country, which would further increase the signal‐to‐noise ratio for arthropods against birds. Arthropods, especially insects, are common in weather radar scans across the UK from late April to early October when warm and dry weather prevails. During this extended period, their aerial abundance generally peaks twice per day: a diurnal peak around midday and a dusk/nocturnal peak in the evening, typically shortly after sunset (Hu et al. 2016). To identify more specific start and end periods for these peaks within a year and within a day, we used annual and diurnal time series profiles of Z_DR_. Data from all 15 WSRs were used to generate two distinct categories of time series profiles: annual time series with a daily resolution and a daily time series with hourly resolution. Using non‐linear Generalized Additive Models (GAMs), we selected a seasonal time window between 15^th^ April to 30^th^ October with peaks in Z_DR_ (corresponding to higher density of horizontally elongated targets, that is, arthropods; Supporting Information Section S6) and truncated the data to only this period for estimating arthropod abundances (Figure S2). Using a similar approach, we identified two different time windows within each day: 0800 to 1400 h and 1800 to 2200 h GMT, corresponding to the maximum in daily Z_DR_ (Figure S2). To avoid repeatedly counting the same insects, we restricted our analysis to a single scan (with maximum Z_DR_) per time window, resulting in two abundance estimates—referred to as diurnal and nocturnal, respectively—per day between 15^th^ April and 30^th^ October. Selecting only one scan per time window also ensures that the unequal temporal coverage of 6 h during diurnal and 4 h during nocturnal does not bias the downstream modeling. The nocturnal scan window may overlap with civil twilight or daylight hours, potentially capturing dusk take‐offs in addition to nocturnal flights. This overlap was accepted to maintain a standardized approach and to capture aerial arthropod abundance in a consistent and comparable manner across latitudes and months.

### 2.4 Estimating Aerial Arthropod Abundance

Columns are approximated as cylinders for the calculation of all mean polarimetric variables at different height bands within a CVP. Therefore, arthropod abundance estimates discussed throughout the text correspond to the volume density within a single “CVP band”, that is, estimated abundance per km^3^ of atmosphere between specific height intervals of 200 m depth and referred to by the lower limit (e.g., abundance density at 500 m corresponds to the mean estimated abundance/km^3^ of atmosphere between 500 and 700 m, and so on).

To estimate abundances at different heights, we adopted the methods developed by Chilson et al. (2012). We converted the radar reflectivity factor (Z) to the more biologically meaningful radar reflectivity (*η*) using the equation: *η* (dB) = Z (dBZ) + *β*, where *β* = 26.58 for the UKMO C‐Band wavelengths (Chilson et al. 2012). The total (mean) reflectivity (in units of decibels) from each height band within a CVP, was then converted to linear units (cm^2^/km^3^), and multiplied by the total volume of a CVP band (km^3^; $V_{h}=\Pi \times r^{2}\times h$, where *r* = 2.5 km and *h* = 0.2 km) to obtain the total back‐scattering area (cm^2^) (i.e., the total reflective surface from all arthropods within a CVP band). By dividing the total back‐scattering area by the estimated mean radar cross section (*σ*) of a single arthropod, we derived the total number of arthropods across different heights (Chilson et al. 2012; Stepanian et al. 2020) (see Supporting Information: Section S2 for more information on how σ was estimated). Dividing this number again by V_h_, we obtained the volume density within a single CVP band. All estimates correspond to the reflectivity from a single radar scan per diurnal and nocturnal time period (the scan with a maximum value of Z_DR_ within each period). This approach avoided double‐counting of individuals that take flights more than once or that remain airborne in the same volume of air over an extended period of time per diurnal or nocturnal time window.

### 2.5 Validation Using Long‐Term Arthropod Monitoring Data

For validation of the estimated abundances, we used concurrent samples from a suction trap maintained by the Rothamsted Insect Survey (Bell et al. 2020), which is within the scan radius of Chenies weather radar (~17.6 km from the suction trap). Using the approach discussed above, we estimated aerial arthropod abundances for different heights above the location of the suction trap. We used Ordinary Least Squares (OLS) regression to assess the relationship between the observed daily arthropod abundances near the ground (from the suction trap data) and the abundance estimates obtained from the CVPs at different heights above the trap.

### 2.6 Statistical Analysis

To model spatio‐temporal variation in aerial arthropod abundance, we focused on estimates from a single band at 500 m, which was represented in the maximum number of CVPs per radar. Lower bands at 100 and 300 m were not available for all CVPs due to radar beam angle (also see Supporting Information: Section S4; results for other heights are discussed in Section S5).

We assessed variation in the aerial arthropod abundance along spatial, temporal, and environmental variables, using a generalized additive modeling (GAM) framework (Wood 2011, 2017). GAM is an additive modeling technique where the impact of the different predictor variables is captured through non‐linear, additive smoothing functions using the general form: $g\left(\mu \right)=\beta +\Sigma_{\left(j=1\right)}^{n}f_{j}\left(x_{j}\right)$, where the mean response (μ) is related to the predictor variables (*x*_1_, …, *x*_n_) by the identity link function *g(μ)* which defines the relationship between the response and ‘*n*’ additive predictors. *β* represents the intercept term, and ƒ_*j*_ is a smoothing function for the predictor *x*_*j*_. Since our estimates of abundance were not derived from individual counts but total reflectivity on a continuous scale, we used Gaussian error distributions to model the estimated abundances instead of the commonly used Poisson for abundance counts. All GAMs were fitted using the R package ‘*mgcv’* (Wood 2011), and the function “*bam*” with discrete = TRUE option for the large dataset.

Using the estimated arthropod abundance densities between 500 and 700 m as the response variable (*μ*), a total of 7 hierarchical spatio‐temporal GAMs were fitted to the diurnal and nocturnal datasets independently (Table S1). The covariates maximum daily temperature (Tmax), Rain, Wind, Artificial Light at Night (ALAN), Elevation, percentage land cover under Arable, Woodland, Grassland, and Urban (built‐up areas + gardens) categories, Year, and the Latitude (*y*) and Longitude (*x*) of each CVP centroid, were fitted with thin‐plate regression splines (Supporting Information: Section S3). As GAMs use shrinkage to reduce overfitting, the predictor “Year” only contributes to the effect not represented by climate and land cover data. This minimizes the probability of wrongly detecting a trend over time that could be attributed to variation in these environmental variables. We included CVP Grid location within the 12 × 12 lattice (Figure 1b), Month, and Radar as random effects. Overall temporal trends in abundance were assessed by using the modelled predictions averaged across all CVPs for each year, while complete spatio‐temporal predictions are based on all significant covariate relationships.

Given the large parameter space, we performed an automated variable selection using the ‘double penalty approach’, implemented via the argument *select = TRUE* in *mgcv*. This approach adds an additional, second penalty that allows shrinkage of the model linear terms, and therefore, when added to the first ‘wiggliness’ penalty, the two can result in an insignificant covariate being entirely removed from the model. The best model was selected using a combination of model diagnostics (normality and spread of the residuals, *k*‐index (Wood 2011), deviance explained, ΔAIC and adj‐R^2^), and AIC scores. We accounted for spatial autocorrelation by including smooth functions of the individual CVP coordinates, that is, *f(x,y)*, and for temporal autocorrelation using AR (1) autoregressive function with the value of the temporal autocorrelation parameter ‘*rho’* estimated using the function *start_value_rho()* from the package *itsadug* (van Rij et al. 2022). Residual spatial autocorrelation (patterns in residuals correlated to spatial proximity) was evaluated using correlograms based on Moran's I (Wood 2003), using CVP centroids as the spatial coordinates. Model fit was evaluated using the *gam.check()* function in *mgcv*.

We used the function *predict.gam(),* which enables a fitted GAM model object to be used for prediction at different values of the model covariates. We also used *predict.gam()* to estimate the (approximate) uncertainty (standard errors) of those predictions obtained by the Taylor expansion approach. These spatio‐temporal predictions were used to generate yearly spatial maps of aerial arthropod abundances per km^3^ of atmosphere. All statistical analyses were performed in the R programming environment (version 4.3.0; R Core Team 2023) on Platform:x86_64‐pc‐linux‐gun (64‐bit). Raw weather data retrieval, storage, and CVP analyses were facilitated using JASMIN, the UK's collaborative data analysis environment (https://jasmin.ac.uk; Lawrence et al. 2013).

## 3 Results

### 3.1 Arthropod Abundance From Weather Surveillance Radars

Median arthropod density within the 500 m CVP band (i.e., abundance/km^3^ between 500 and 700 m height) was 4.61 × 10^7^ (interquartile range = 3.77 × 10^8^) and 2.06 × 10^7^ (interquartile range = 2.91 × 10^8^) diurnal and nocturnal arthropods, respectively. Extrapolating this to the entire UK indicates that an average of 1.12 (±0.01) × 10^13^ diurnal and 5.02 (±0.01) × 10^12^ nocturnal arthropods were present over the UK between 500 and 700 m height, between 15th April and 30th October, and at any given instance between 0800–1400 and 1800–2200 GMT, respectively, although with high inter‐annual variability (Figure S3).

On average, arthropod abundances decreased monotonically at the rate of 8.74 (±0.01) × 10^5^ individuals per 200 m of height gained in the air column (Diurnal: slope = −7.77 (±0.01) × 10^5^, *Adj. R* ^*2*^ *= 0.11, p < 0.001*; Nocturnal: slope = −9.71 (±0.21) × 10^5^, *Adj. R* ^*2*^ *= 0.12, p < 0.001*; Figure S4).

### 3.2 Validation Using Long‐Term Arthropod Monitoring Data

**FIGURE 2:**
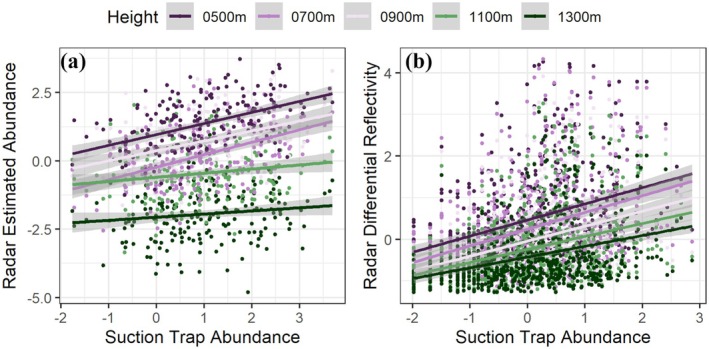
Validation of radar‐derived estimates of arthropod abundance using a ground‐based suction trap. We obtained daily total arthropod counts from a 12.2 m suction trap maintained by the Rothamsted Insect Survey, which is 17.6 km from the Chenies weather radar, and therefore within the radar's scanning range. We estimated aerial arthropod abundances for different heights in the air column, at the location of the suction trap using the methods developed in this study. We used Ordinary Least Squares (OLS) regression to assess the correlation between the observed daily arthropod abundances near the ground (from the suction trap data), and the abundance estimates obtained from the CVPs at different heights. We found strong and significant correlations between the observed arthropod abundance recorded at the suction trap, and (a) abundance estimated from the Chenies weather radar, and (b) Z_DR_ or differential reflectivity. We measured the correlations at different heights within the CVP and observed that the slope of both relationships decreased with height, with the strongest relationship at the lowest height. We used scaled variables for regression models since the two datasets are obtained at different spatial scales.

Based on the dual‐polarization coverage of the Chenies WSR and the number of operational days at the Rothamsted suction trap, we obtained *n* = 127 days that overlapped across the two datasets. We further removed days (*n* = 9; entire day, i.e., 24‐h removed) where heavy rainfall occurred, resulting in a total of 116 days for comparison. We found strong and significant correlations between estimated abundances and at different heights in the CVP with the observed arthropod abundances at 12.2 m suction traps (*Adj. R* ^*2*^ = *0.32* to *0.47*; *p* < *0.001*; Figure 2a). As expected, the slope of this relationship decreased with height, with the strongest relationship at the lowest height (Figure 2a).

### 3.3 Spatio‐Temporal Variation

Of the 7 hierarchical GAMs tested (Table S1), the best fitting model included the following terms:

$$\begin{array}{c} g\left(\mu \right)=f1\left(\text{year}\right)+f2\left(\mathrm{yea}r_{f},R\right)+f3\left(\text{radar},R\right)+f4\left(\text{Year},\mathrm{by}=\text{Radar}\right)\\ +f5\left(\text{month},R\right)+f6\left(\mathrm{CV}P_{\text{location}},R\right)+f7\left(x,y\right) \end{array}$$

along with the following 9 covariates:

$$\begin{array}{c} f8\left(T_{\max}\right)+f9\left(\text{Rain}\right)+f10\left(\text{Wind}\right)+f11\left(\text{Arable}\right)+f12\left(\text{Urban}\right)\\ +f13\left(\text{Woodland}\right)+f14\left(\text{Grassland}\right)+f15\left(\text{ALAN}\right)+f16\left(\text{Elevation}\right) \end{array}$$

**FIGURE 3:**
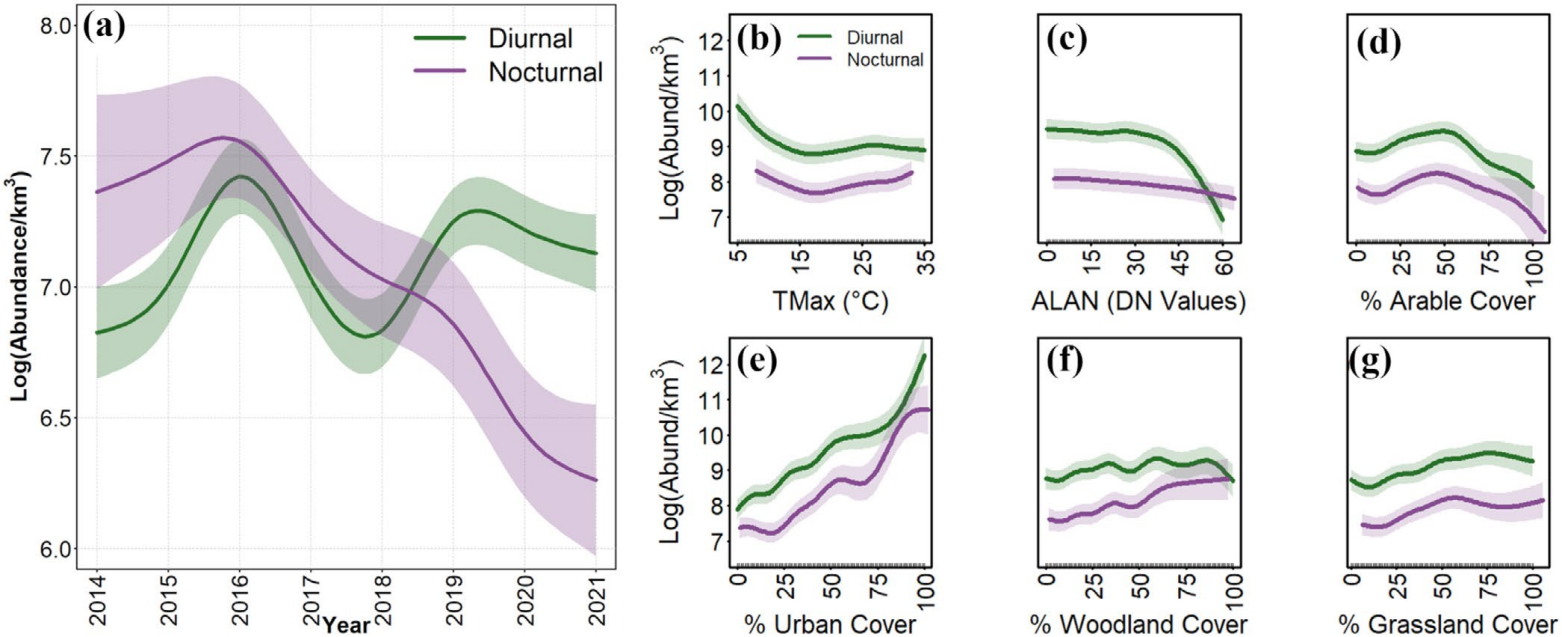
Temporal trends and drivers of variation for aerial diurnal and nocturnal arthropod abundances estimated over 35,000 sq. km in the UK, using UK‐Met Office weather radar stations across an 8‐year period. Within each plot, the values on the *y*‐axis correspond to arthropod abundance per km^3^ between 500 and 700 m in the atmosphere (a) Cumulative abundances for diurnal (between 0800 and 1400 GMT; *shown in green*) and nocturnal (between 1800 and 2200 GMT; *shown in purple*) aerial arthropods were predicted using generalized additive models for each year between 2014 and 2021 (for raw temporal series see Figure S3) (b–g) Each plot shows a covariate on the *x*‐axis and aerial arthropod abundance on the *y*‐axis. Variables shown are (b) TMax: Maximum daily air temperature; (c) ALAN: Artificial Light at Night measured using DN Values that is, Digital Number, which ranges from 0 to 63, where 63 represents maximum night‐time illuminated sky; (d–g) Percentage land cover under arable, urban, woodland and grassland. The relationships are shown for both diurnal (green) and nocturnal (purple) arthropods.

This model explained 80.2% and 76.4% of the total deviance in diurnal and nocturnal arthropods respectively and revealed significant spatio‐temporal heterogeneity across the WSR network (Tables S2 and S4). Average cumulative predictions per year revealed significant declines in nocturnal arthropod abundances over time; however, diurnal abundances did not exhibit a consistent negative trend with year (Figure 3a). Nearly all the tested variables had similar patterns of associations with both diurnal and nocturnal arthropod populations, indicating a broad‐ scale generality of the relationships (Figure 3b–g). The only variable showing different effects on diurnal and nocturnal arthropods was ALAN, which had a weak negative effect on nocturnal species, and a strong negative effect on diurnal ones, but only at higher ALAN levels. Woodland and grassland cover had positive associations (Figures 3g and 3f), while arable cover revealed a negative relationship with aerial arthropod abundances but only for high arable land cover (Figure 3d). Across the individual, height‐stratified GAMs, the estimated effect sizes (and significance) of land cover covariates declined progressively with increasing height (Supporting Information: Section S5).

**FIGURE 4:**
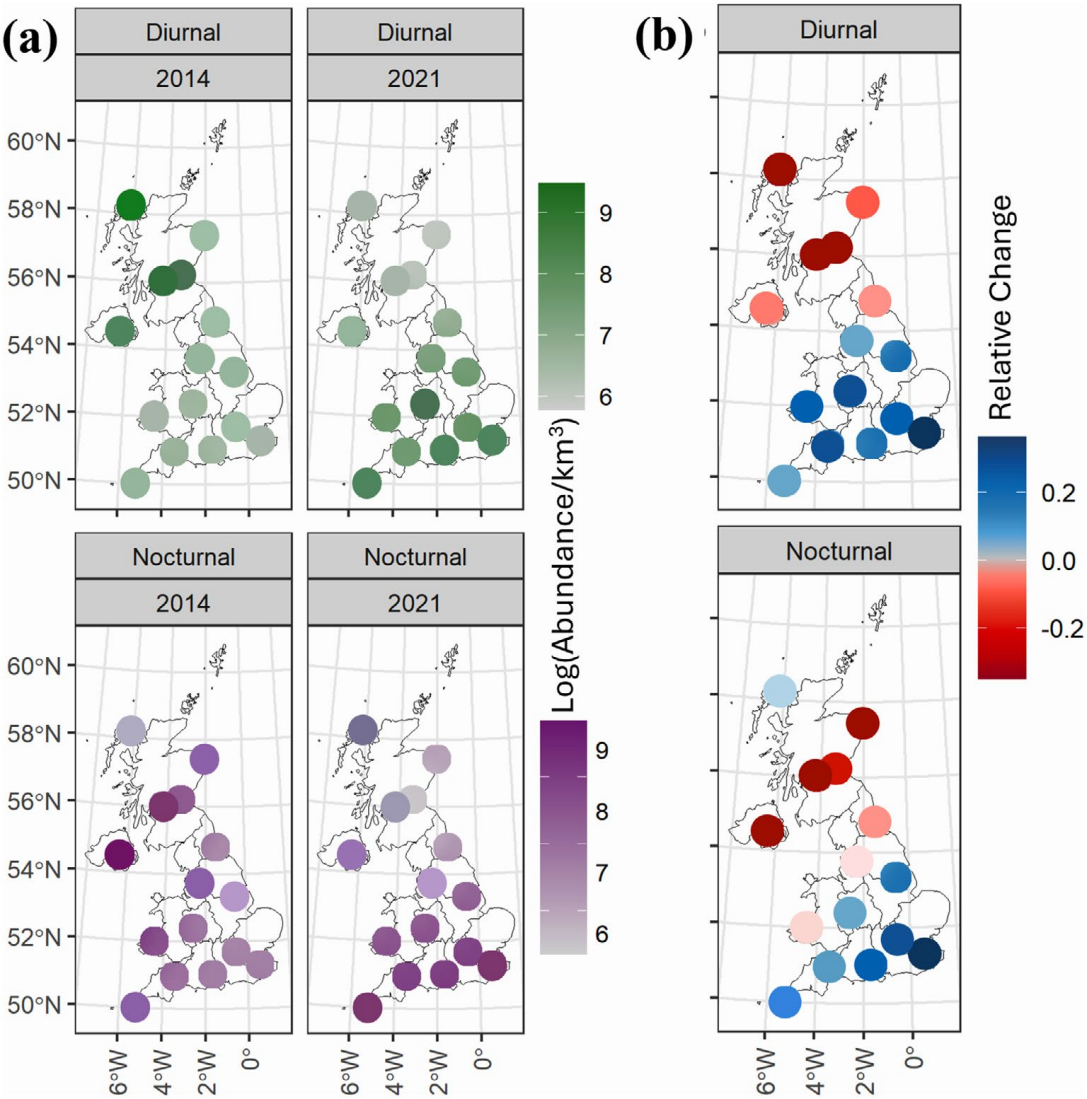
Spatio‐temporal surfaces for diurnal (top) and nocturnal (bottom) aerial arthropod abundance estimated from UK‐Met Office weather radar stations across an 8‐year period in the UK. The shaded circles overlap the 15 UK weather radars for which dual‐polarized data was available. Aerial arthropod abundances were estimated for approximately 127 Columnar Vertical Profiles (a cylindrical volume of atmosphere, 2.5 km in radius and roughly spanning 1.8 km in height between 100 and 2100 m) around each radar (the shaded circles shown above are slightly enlarged for clarity). Generalized Additive Model (GAM) was used to model the spatio‐temporal relationships between abundances (only between 500 and 700 m) and latitude, longitude and year. (a) Shown here are the model outputs for only 2014 and 2021 for diurnal (top) and nocturnal (bottom) aerial arthropod abundances. (b) Corresponds to the relative change from 2014 to 2021, with negative values indicating a decline in log (abundance/km^3^) of aerial arthropods. Map lines delineate study areas and do not necessarily depict accepted national boundaries.

**FIGURE 5:**
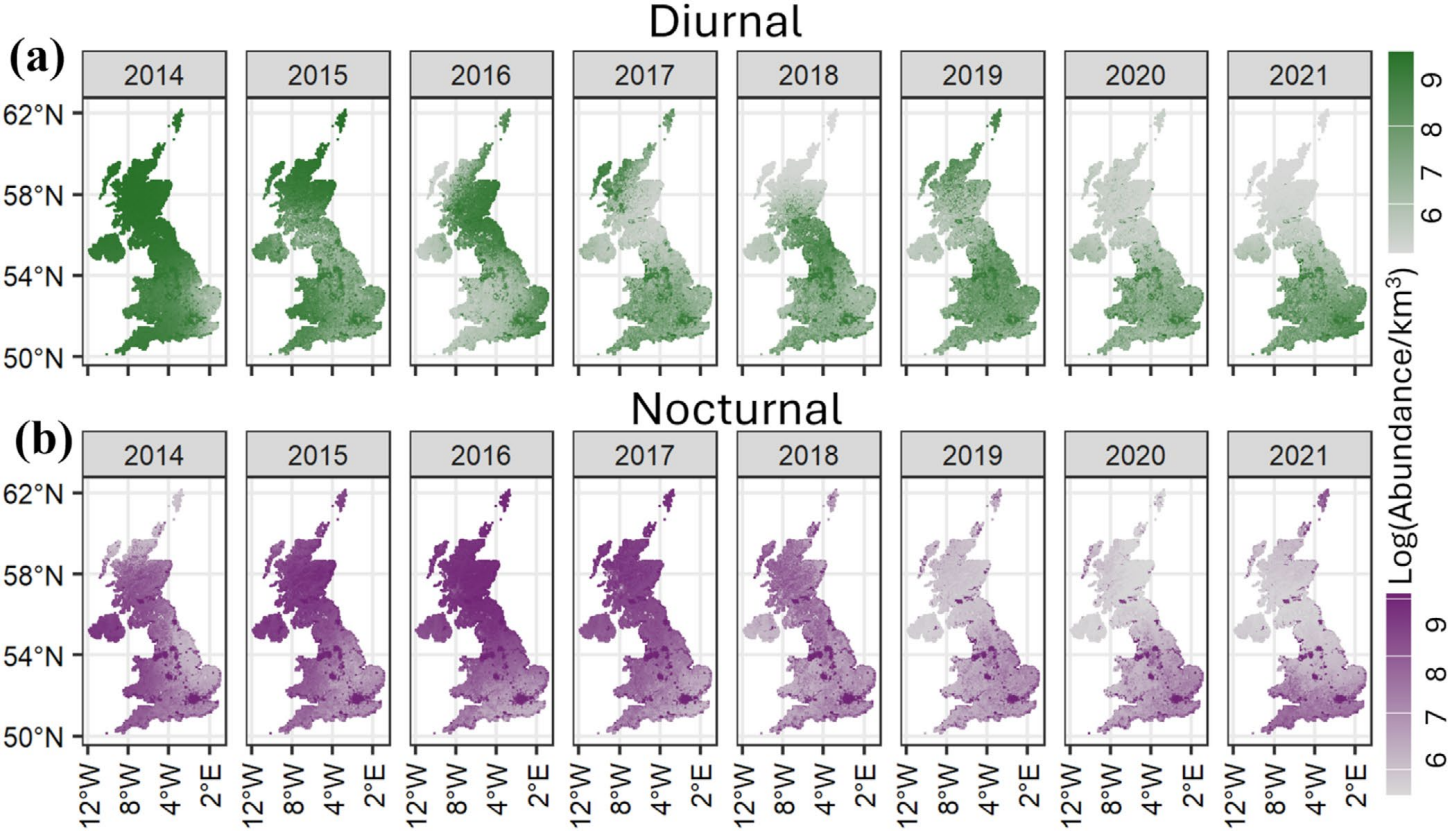
Predicted abundance densities of diurnal (shown in green; top row) and nocturnal (shown in purple; bottom row) aerial arthropods between 500 and 700 m height in the atmosphere, across the UK between 2014 and 2021. The model predictions across the entire country are derived by combining the stacked rasters of underlying covariates such as weather, land cover, elevation, and artificial light at night (ALAN), and using the modelled relationships between these covariates and arthropod abundance (as shown in Figures 3 and 4). Map lines delineate study areas and do not necessarily depict accepted national boundaries.

Arthropod abundances showed a strong spatial dependence, with a significant effect of the smoothed terms for the CVP's × and *y* coordinates [*f*_*7*_ *(x,y)*]; the temporal trends exhibited a higher net decline towards the higher latitudes for both diurnal and nocturnal arthropods (Figure 4). We also observed an increase (positive change) in arthropod abundances at the lower latitudes (Figure 4). The modelled relationship between abundance and all covariates was used to generate national‐scale spatio‐temporal predictions for new, unsampled locations (Figure 5).

## 4 Discussion

By employing an extensive and standardized dataset on a national scale, our study has revealed important broad‐scale spatio‐temporal patterns in the abundance of aerial arthropods across the UK between 2014 and 2021. On average, nocturnal arthropods showed a decline in abundance, while diurnal arthropods showed substantial inter‐annual variation, but no overall increasing or decreasing trend (Figure 3a). However, these trends were not consistent across all regions; both groups exhibited significant increases in abundance over the southern latitudes, with declines primarily confined to the northernmost regions (Figure 4). Our study emphasizes the significance of spatial variation in obscuring temporal trends (Wagner et al. 2021), which is likely important when analyzing the impact of spatially structured drivers. Furthermore, we have demonstrated that WSR networks can deliver systematic, non‐invasive biodiversity monitoring, which provides large‐scale and continuous coverage at high temporal resolutions.

Spatio‐temporal variation indicated declines in arthropod abundance at higher latitudes across the UK, compared to the south (Figure 4b). The decline in the north reflects the observed negative association between maximum daily air temperature (T_max_) and arthropod abundances, which were most prominent at lower values of Tmax typical of northern latitudes in the UK (Figure 3b). Temperature has increased in the UK over the study period (Christidis et al. 2023), and the positive correlation between arthropod abundance and Tmax at higher values of the latter would also explain the increase in the southern latitudes. Recent warming has been highly uneven across the globe, with higher latitudes warming faster than the tropics (Intergovernmental Panel on Climate Change (IPCC) 2021). However, the UK Climate Projections 2018(UKCP18) projections reveal the opposite latitudinal gradient for the UK: maximum temperatures have risen (and are projected to rise) more sharply in southern England than in northern Scotland (Lowe et al. 2018; Murphy et al. 2020). This north–south asymmetry in warming, together with the positive correlation between arthropod abundance and higher Tmax, would offer some explanation for why increases were concentrated in southern CVPs, whereas declines were largely confined to the northernmost regions. These findings underscore how spatial variation in climate change can drive contrasting temporal biodiversity trends within a relatively small geographic area. Previous research has shown that distinct atmospheric layers in aerial arthropods are associated with local maxima in the vertical air temperature profile (Drake 1984; Wood et al. 2006), suggesting that the inclusion of finer‐scale variables (vertical profiles of local climate) is likely to improve the prediction of aerial arthropod variability in radar datasets in future (e.g., UK Met Office's numerical weather prediction model, the “Unified Model”) (Brown et al. 2008).

Habitat type and land cover changes have been identified in the past as the main drivers of arthropod declines, a factor implicated equally in global bird and mammal declines (Chamberlain and Fuller 2000). While our samples are constrained to arthropods suspended in the atmosphere above the habitat matrix below, we did find associations with the different habitat types. We observed a negative relationship of aerial arthropod abundances with arable cover, and a positive relationship with woodland, grassland, and, surprisingly, urban land cover. The negative effects of increasing arable cover are often mediated by loss of native plants, increased use of pesticides and fertilizers, increased frequency of harvest in recent years, and others, which are deemed to be key drivers of arthropod declines (Fox 2013). The strong positive effect of urban cover (Figure 3e) may be due to urban heat island effects (Youngsteadt et al. 2017); arthropod aerial movements, particularly at higher heights, are triggered by steadily rising isothermal currents associated with warmer temperatures of urbanized regions (Reynolds et al. 2008). A similar observation was noted recently for birds (Van Doren et al. 2017). Although the pattern is contrary to expectation, it should be noted that ‘urban cover’ represents a broad, heterogeneous category spanning all built‐up areas, gardens, and suburban areas. Thus, a more detailed investigation into the relative abundances across these categories may provide a deeper understanding of the role of urban cover on aerial arthropod abundances. This positive association likely causes predictive modeling to show urban regions as the most prominent hotspots of aerial arthropod abundance across the UK (Figure 5).

The predicted patterns of urban insect abundance differed markedly between nocturnal and diurnal arthropods, with nocturnal densities elevated throughout urban areas, while diurnal taxa showed depressed abundance in urban centers. This suggests that the concentration of nocturnal arthropods in cities could at least partly be due to the attraction to ALAN, as shown previously for birds (Van Doren et al. 2017) and insects (Tielens et al. 2021). For example, urban areas of Las Vegas (USA) were previously characterized as a large‐scale attractive sink on nocturnal flights of arthropod populations, indicating the attractive or disorienting effect of artificial light (Tielens et al. 2021). ALAN impacts the vital biological functions of nocturnal and diurnal arthropods alike; it alters the circadian patterns of activity and rest in diurnal arthropods, which results in impaired immune function, reduced fecundity, and a shorter lifespan (Durrant et al. 2015; Kouser et al. 2014). It also causes diurnal and crepuscular arthropods to move their foraging activity into the night, which subjects them to increased predation (Garber 1978), and cold stress (Owens and Lewis 2018). Despite a potentially negative effect on both nocturnal and diurnal arthropod populations, the impact on nocturnal arthropods may be masked by positive density effects due to behavioral attraction; nocturnal arthropods are drawn to light sources across larger distances (Owens and Lewis 2018). On the other hand, the negative fitness effects on demography should accumulate over time via effects on arthropod circadian rhythms, navigation, and foraging behavior (Manfrin et al. 2017). The stronger negative effect of very high ALAN values on diurnal arthropods in our findings is counterintuitive (Figure 3c) and may be due to some other driving variable not considered in the present analyses. Specifically, the very high ALAN intensities associated with reduced diurnal arthropod abundances may be associated with core cities, and/or with industrial or transport infrastructure, distinguishing them from suburban environments characterized by only moderate ALAN levels. With temporal niche partitioning between diurnal and nocturnal species becoming less extreme in response to human activity (Levy et al. 2019; Owens et al. 2020), more research is needed to document the role of ALAN in arthropod declines, including diurnal groups/species. We ensured that the diurnal effect of ALAN was independent of urban cover by re‐running our models after accounting for the correlation between ALAN and urban cover (Supporting Information Section S4).

As previously mentioned, all spatio‐temporal patterns and predictions discussed here correspond to the arthropods within a specific height band in the atmosphere (between 500 and 700 m). Previous work has shown that the median flight layer has remained altitudinally stable over the past decade (Gao et al. 2020), and that there is strong temporal coupling among neighboring (vertically adjacent) layers (Reynolds et al. 2005). These observations suggest that a single, broad altitudinal band provides a reliable index of (relative) spatio‐temporal changes in aerial abundances of arthropods. Although the vertical layering is strongly governed by temperature inversions, boundary‐layer depth, and wind shear (Drake 1984; Reynolds et al. 2005), these phenomena have so far reported weak or non‐monotonic long‐term trends in previous studies (Shahi et al. 2020; Yue et al. 2021; Zhang et al. 2013). Nevertheless, future work linking height‐resolved arthropod abundances with detailed, local temperature profiles and atmospheric processes will be essential to detect climate‐ and habitat‐driven redistribution of flight heights. We analyzed the land‐cover relationship for estimated arthropod abundances at different heights and observed a diminishing influence of land cover variables with increasing height (Supporting Information: Section S5). Notably, aerial arthropods at heights greater than 900 m were not significantly correlated to a single land cover variable. This indicates that arthropods undertaking flights at higher heights are decoupled from the underlying habitat type, most likely because they are engaged in a long distance flight, covering distances greater than our CVP spatial resolution. This is further supported by the large number of recent studies showing that even the tiniest aerial arthropods (e.g., aphids and micro‐hymenopterans) are not entirely passive in their dispersal processes (Bell and Shephard 2024; Ortega‐Jiménez and Combes 2018; Reynolds and Reynolds 2009; Wainwright et al. 2017), and exhibit attraction to light sources (Döring and Chittka 2007; Kirchner et al. 2005). Future studies are needed to delve deeper into the size and taxonomic classifications of radar observations, providing clearer insights into how spatio‐temporal trends translate to different ecological groups (Lukach et al. 2022).

Much of our macroscale understanding of arthropod diversity trends so far has been derived from studies on ground‐dwelling and/or low‐flying diurnal insects. Consequently, it is not unexpected that some of the emerging results—especially the positive association between urban land cover and aerial arthropod density, and the negative effect of ALAN on diurnal arthropods—are novel and counterintuitive. These observations show that aerial arthropods may not be temporally and/or spatially synchronized with arthropod activity at ground level and hence may not accord with the monitoring of field‐caught species or the perceptions of those who collect them. It is also the case that these arthropods are almost entirely monitored during one life stage—the adult winged phase, part of a much more complex life cycle that cannot be measured using radar. The importance of this study is to open a window to a huge and important new source of biodiversity monitoring data. Our findings here are just a tantalizing glimpse of what such data can reveal, and further, long‐term analyses should be conducted as these datasets grow longer, especially to confirm the continuity of the temporal trends we detect.

Our work has provided significant insights into aerial arthropod activity, confirming and extending findings initially observed with Vertical Looking Radars (VLRs; Supporting Information Section S6). For instance, the positive correlation between differential reflectivity (Z_DR_) and aerial arthropod density (Figure 2b) is consistent with VLR observations of horizontally aligned targets at similar heights. Peaks in Z_DR_ between April and October, and during mid‐day and evening, also validate earlier observations of high insect activity during these windows (Hu et al. 2016). The extensive scale of our results reveals the broad‐scale generality of these mechanisms across a range of biomes.

A series of interesting research gaps emerge from our work. First, the taxonomic and/or morphological resolution that can be derived from WSR observations requires further analysis. Although current radar‐based estimates of arthropod abundance are not species‐specific (Bauer et al. 2024; Chapman et al. 2011; Gauthreaux and Diehl 2020; Hüppop et al. 2019), recent studies suggest that WSR data—especially when coupled with ground‐based monitoring—have the potential to discriminate among different biological taxa, at least at higher taxonomic levels (e.g., Orders) (Hu et al. 2024; Lukach et al. 2022). There is a need for extensive work in electromagnetic modeling and simulation to explore radar cross sections of a diverse array of arthropod taxa to classify the radar data by broad taxonomic groups (Matthews et al. (in press); Addison et al. 2022; Mirkovic et al. 2016, 2019). Our analyses here have assessed only overall arthropod numbers, but a degree of morphological information concerning sizes and shapes is provided in dual‐polarization radar reflectance data. Future studies could be explicitly designed to bridge the gap between ground‐based long‐term monitoring and weather radar observations; high‐throughput tools such as metabarcoding from suction trap samples, along with strategic new sampling approaches (e.g., drone‐based aerial surveys), could help build the crucial taxonomic link between radar signals and biological identity. Second, much of the research using radar has focused on migratory organisms rather than resident populations. The relative contribution of migrants to local arthropod communities, and, hence, the value of migration to the ecosystem services that are provided by those communities remains poorly understood. Incorporating data from citizen and community scientists, who increasingly contribute to species‐level occurrence data and can measure near‐ground abundances that are invisible to the WSR, particularly for moths and freshwater insects migrating along watercourses in the UK, could enhance our understanding of local arthropod communities and their ecological contributions. Addressing these issues will require collaborations between scientists, engineers, conservation practitioners, policymakers, and citizen scientists to advance the use of radar‐derived measures in biodiversity conservation.

Our research is one of the first studies to empirically assess changes in abundance, and their potential drivers, across a broad spectrum of aerial arthropod taxa at a national scale. Spatial heterogeneity has posed a significant challenge in reconciling temporal trends in arthropod declines, even within a single taxonomic group (Didham et al. 2020). Until now, it has remained uncertain whether observed heterogeneity stemmed from methodological disparities between studies or was an inherent characteristic of arthropod communities (Wagner 2020). The methods developed herein provide insights into both diurnal and nocturnal arthropod trends using a single monitoring method, something that is missing from contemporary monitoring methods. This analytical framework can be used to investigate how future changes in major environmental conditions may influence aerial arthropod densities. This is the first critical step for better understanding their roles in ecosystem functions and services.

The benefits of WSR observations come at relatively little marginal cost because the underlying infrastructure—comprising radar installations, data acquisition systems, and archival platforms—is already established and maintained through national meteorological services for operational weather forecasting. Unlike traditional arthropod monitoring methods, which often involve resource‐intensive collection tools and incur significant field costs for data collection, weather radar data are continuously and passively collected at high spatio‐temporal resolution. The primary costs associated with the ecological use of radar data arise not from data acquisition but from data processing. These include maintenance of processing scripts and pipelines (1 person‐month per year; ~£10,000 with full economic costing), storage and compute capacity (estimated at £10,000 to £30,000 annually depending on data volume and archival depth, though currently subsidized for NERC projects via platforms such as JASMIN), and updates to classification algorithms in response to changes in radar hardware or improvements in methodology (additional personnel; at approximately £10,000 per year). These are best viewed as fixed service‐level costs, akin to community‐wide resources like GBIF or GenBank, rather than project‐specific expenses. Given the ubiquity of existing national WSR networks across Eurasia, the Americas, and Australasia (as well as the current expansion of networks globally), there are exciting prospects for continental or even global‐scale biodiversity monitoring in the future.

## Author Contributions

**Mansi Mungee:** data curation, formal analysis, methodology, software, visualization, writing – original draft, writing – review and editing. **Maryna Lukach:** methodology, visualization, writing – review and editing. **Chris Shortall:** methodology, validation, writing – review and editing. **James R. Bell:** conceptualization, funding acquisition, methodology, project administration, supervision, writing – review and editing. **Elizabeth J. Duncan:** conceptualization, funding acquisition, writing – review and editing. **Freya I. Addison:** writing – review and editing. **Lee E. Brown:** funding acquisition, methodology, project administration, supervision, writing – review and editing. **William E. Kunin:** conceptualization, funding acquisition, investigation, methodology, project administration, supervision, writing – review and editing. **Christopher Hassall:** conceptualization, funding acquisition, investigation, methodology, project administration, supervision, writing – original draft, writing – review and editing. **Ryan R. Neely III:** conceptualization, data curation, funding acquisition, investigation, project administration, resources, software, supervision, writing – review and editing.

## Conflicts of Interest

The authors declare no conflicts of interest.

## Supporting information


**Data S1:** gcb70425‐sup‐0001‐Supinfo1.pdf.

**Data S2:** gcb70425‐sup‐0002‐Supinfo2.pdf.

## Data Availability

The data and code that support the findings of this study are openly available in Zenodo at https://doi.org/10.5281/zenodo.16418783. WSR data were obtained from the Centre for Environmental Data Analysis (CEDA) Archive at http://catalogue.ceda.ac.uk/uuid/82adec1f896af6169112d09cc1174499.
