# Supplementary material for: Spatio‐Temporal Variation in Aerial Arthropod Abundance Revealed by Weather Radars
Source: Glob Chang Biol. 2025 Oct 27;31(10):e70425. doi: 10.1111/gcb.70425 (PMC12555869; doi:10.1111/gcb.70425)
Supplement: Supplementary file 1 — Data S1: gcb70425‐sup‐0001‐Supinfo1.pdf. [file GCB-31-e70425-s001.pdf]

Supporting Information

Spatio-temporal Variation in Aerial Arthropod Abundance Revealed by Weather Radars

Authors: Mansi Mungee, Maryna Lukach, Chris Shortall, James R. Bell, Elizabeth J. Duncan, Freya Addison, Lee E. Brown, William E. Kunin, Christopher Hassall, Ryan R. Neely III*

***Corresponding author:** Ryan R. Neely III
Email: R.Neely@leeds.ac.uk

This PDF file includes:

S1. Columnar Vertical Profiles (CVPs): Page 3 - 4

S2. Radar Cross Section: Page 4 - 5

S3. Environmental covariates: Page 5 - 7

S4 Generalized Additive Models (GAMs): Page 7 -8

S5. GAMs at individual height bands: Page 8 - 9

S6. Comparison with VLRs: Page 9 - 10

References: Page 11 - 13

Figures S1 to S10: Pages 15 - 24

Tables S1 to S7: Pages 25 - 31

S1. Columnar Vertical Profiles (CVPs)

The UK Met Office radar observations utilize five elevation angles (ranging from 0.5° to 4.0°) and 360 azimuthal directions (with 1° resolution) across 600 m equidistant range gates, extending out to 255 km. This scanning geometry captures a volume of the atmosphere surrounding each radar, consisting of volume pixels—voxels—assigned to spherical coordinates.

Throughout this text, we use the term "elevation angle" when referring to radar volume scans (e.g., "observations from the lowest elevation angle of a radar volume scan" or "PPI of the 4-degree elevation angle"). The "height band" or "height" of a CVP band corresponds to "above ground level (AGL)" and is calculated based on assumed refraction, scanning geometry, and the estimated curvature of the Earth. We use "depth" to describe the vertical step size of the different height bands in a CVP.

The generation of CVP begins by detecting all the voxel centres within a specified CVP radius. Data from multiple elevation scans within the 600 m × 1° sector are azimuthally averaged and projected to the CVP centre, producing a vertical profile of voxels. The voxel detection process follows the method outlined by Murphy et al.[Main ref. no. 23]. Briefly, a centre voxel is selected from the radar's spherical grid closest to the column's geodetic coordinates at the lowest elevation. This centre is then projected onto other elevations of the volume scan along a constant bearing and range path. An azimuthal delta is calculated for each column centre based on the size of the centre voxel, compensating for the azimuthal broadening of the radar beam with range. The range delta depends only on the range resolution of the radar measurements and the column size; thus, it is constant for all columns. All voxels whose centres fall within these azimuthal and range deltas are considered column voxels. For each dual-polarization variable, a mean value is assigned to the corresponding height band, calculated from all voxels with valid (non-NaN) values whose centres fall within that band. These mean values are then assigned as the profile value for that height.

Although, technically, columns are not perfectly circular nor strictly vertical, we selected a circular representation for simplicity and consistency in our calculations. This representation is used for all visualizations and column volume estimates. Cylindrical columns can be viewed as the closest approximation of a volume comprising a subset of voxels participating in the mean calculation. The CVP values, therefore, represent a mean across the available radar observations at a specific point within a given radius.

We generated CVP columns with a radius of 2.5 km and a vertical resolution (step size or depth for each height band) of 200 m (see Fig. 1b for the distribution of columns relative to the radar). This radius and step size were chosen based on a trade-off between column diameter (sector size) and projection limits, ensuring uniform data averaging and projection. We tested different diameters

and resolutions of the step sizes, analysing 114 combinations (not shown) with diameters ranging from 1 km to 10 km, each with 50–300 m bands (with a bin size of 50 m). The optimal CVP diameter was selected as the smallest possible that still provided a high number of mean-per-height-layer values across different heights.

Following this approach, arthropod abundance estimates in this study correspond to the volume density within a single CVP band—i.e., estimated abundance per km³ of atmosphere—between specific height intervals of 200 m depth, referred to by the lower limit (e.g., the abundance density at 500 m corresponds to the mean estimated abundance/km³ of atmosphere between 500 and 699 m, and so forth).

We calculated 144 CVPs for 15 radars across the UK by generating a 60 km × 60 km lattice (spatial bounding box) with each radar's spatial coordinates as the centroid (Fig. 1). Since the UKMO radars are spaced more than 60 km apart, this configuration allowed us to obtain distinct, non-overlapping datasets while preserving the independence of data collected from each radar site. The 60 km × 60 km lattice was populated with a 12 × 12 CVP arrangement in a regular grid, resulting in a total of 144 × 15 = 2,160 CVPs across all radars (Fig. 1b). However, because the radar beam height is a function of range (distance from the radar), we removed 16 central CVPs from the 12 × 12 grid where the beam height—and consequently, the number of voxels within the 500 m height band—was very low. Although this resulted in some data loss, it did not bias our results as the same locations were affected across all radars, and the number of CVPs per radar remained constant. In Fig. 1b, we illustrate our grid approach, including the missing CVPs. Additionally, due to technical issues, one CVP could not be processed (the same CVP in the upper-right corner of Fig. 1b for all radars), resulting in 127 CVPs per radar.

We excluded all CVPs where radar beam obstructions could lead to significant ground clutter. To do this, we identified potential obstructions within a 60 km × 60 km area around each radar using a 90m Digital Terrain Model (DTM)[1,2]. For each CVP, we extracted DTM cell values along the radar beam path connecting the CVP's coordinates to the radar. If any cell value along this path exceeded the radar beam's lowest limit (i.e., if $\text{Elevation}_{\text{DTM}} > \text{Elevation}_{\text{BEAM}}$, accounting for beam width[1]), we removed that CVP from further analysis. This method effectively identifies areas where terrain exceeds the radar beam's lowest elevation, eliminating affected CVPs. However, we acknowledge that this approach does not fully account for shadowing or echoes around and beyond the CVP.

S2. Radar Cross Section

Radar Cross Section (RCS) (σ) is a measure of a target's ability to reflect or scatter electromagnetic waves back to a radar system. The RCS value depends on several factors,

including the target's size, shape, composition, and orientation relative to the radar beam. As a result, a single RCS value is unlikely to represent the wide variety of arthropods typically detected by weather radars. However, during the calculation of CVPs, the different azimuthal dependencies of σ are averaged out, and therefore it can be potentially expressed as a single value.

Riley (1975)[3] demonstrated that the radar cross-section of an arthropod could be approximated by that of a spherical water droplet of equivalent mass. This approximation holds across a mass range from 10 mg (aphids) to 1,000 mg (grasshoppers) for X-band radar, with a wavelength (λ) of 3.2 cm. More recently, advancements in experimental microwave anechoic chambers and mathematical models have further refined this relationship for different wavelengths including at C-Band[4-7].

In a study using Vertical Looking Radar (VLR) and aerial netting across southern UK, Hu et al. (2016) reported that over 99% of aerial arthropods are small (<10 mg, primarily from the Aphidoidea and Diptera groups), while the 15 billion medium and 1.5 billion large arthropods comprised only 0.4% and 0.05% of the annual abundance, respectively. Since our study focuses on abundance rather than biomass, we chose to consider arthropods smaller than 10 mg, corresponding to an average body length of 1.5 mm. Given that the wavelength of UKMO radars is 5.3 cm, nearly 100% of our target group is smaller than the radar wavelength, meaning Rayleigh scattering applies in most cases. Based on the scaling relationship from previous research[3-6], we 123 estimate an average σ of 0.000452 cm^2, which represents over 99% of UK arthropods.

We acknowledge that our σ estimates are conservative and closer to the lower bound of 126 abundance estimates derived from reflectivity. We assume that arthropod density is dominated by a large number of small arthropods, a notion supported by the high accuracy and precision reported for 1.5–2 mm drop-size distributions from C-band radar measurements of precipitation[8-9]. This suggests that even small arthropod targets are detectable. Further studies are needed to assess the effects of dielectric constant and shape distribution on detection accuracy for arthropods of different sizes. However, any increase in RCS value is unlikely to impact the overall spatio-temporal patterns derived from estimated abundances or alter the relative values across CVPs, though it may influence the absolute values of the estimates.

S3. Environmental covariates

We curated an initial set of 15 environmental variables known to influence arthropod populations.

- **5 weather variables:** three temperature variables (surface mean, minimum and maximum daily temperatures; TMean, TMin, and TMax respectively), daily precipitation, and daily average surface-wind speeds were compiled from the HadUK-Grid gridded datasets at 1km resolution archived at the Centre for Environmental Analysis[10].

- Altitude above mean sea level was obtained from a 90m Digital Terrain Model (DTM)[2].
- **Artificial Lights At Night (ALAN)** was obtained from the harmonized global nighttime light dataset (1992 - 2018) which provides a global coverage of 'DN values'[11]. DN values, representing illumination, are recorded from satellites as the number of 'lit pixels', on a linear scale from 0 (dark) to 63 (instrument light saturation). Saturation occurs when pixels in bright areas, such as in city centers, reach the highest possible digital number value (i.e., 63) and no further details can be recognized. This data excludes observations affected by stray light and corrects for the effects caused by bio geophysical processes such as seasonal dynamics of vegetation and snow using the bidirectional reflectance distribution function[11]. The ALAN dataset was available at yearly resolution.
- **Seven landscape variables** were compiled from the UK-Centre for Ecology and Hydrology (CEH) Land Cover Maps (LCM) Series[12-19]. LCM series are created by classifying satellite data into 21 land cover classes, which are based on the UK Biodiversity Action Plan Broad Habitat definitions (Jackson, 2000). We used the UK-CEH Land-cover maps (2007, 2015, 2017, 2018, 2019, 2020 and 2021 editions[12-19]) to derive a time series between 2014 and 2021 using the function *na_interpolation()* from R package imputeTS[20]. Percentage land cover under arable, woodland, grassland, urban, mountains, coastal, saltwater and freshwater were extracted for each CVP. Although further sub-classes are available, here we have used only broad, aggregated classes as follows:
    - *Arable: arable and horticulture*
    - *Woodland: broad-leaved, mixed, coniferous and yew*
    - *Grassland: improved and semi-improved (neutral, calcareous, acid grassland, fen, marsh and swamp)*
    - *Urban: urban and semi-urban (built-up areas and gardens)*
    - *Mountains: dwarf shrub heath, heather, heather grassland, bog, inland rock*
    - *Coastal: supra littoral rock, supra littoral sediment, littoral rock, littoral sediment and saltmarsh*
    - *Freshwater*
    - *Saltwater*

All variables were aggregated at 5km to match the spatial resolution of CVPs. To assess multicollinearity, we used Pearson's correlation coefficients and a Variance Inflation Factor (VIF) analysis. We removed variables which were strongly correlated (Fig. S5). We additionally removed four land cover categories (mountains, coastal, freshwater and saltwater), which were very sparsely represented in many CVPs (<10% CVPs had freshwater cover greater than 10% of the CVP area, while 95 % CVPs contained 0% salt-water; Table S7). These variables provided almost no information for most sites. Thus, when entered as smooth terms, their spline bases collapsed for the majority of observations, producing high concurvity and very large standard errors; in

several runs the REML optimiser failed to converge, and we therefore removed these variables from our final models.

The pair-wise correlations among the final set of shortlisted predictors ranges from -0.02 to 0.47, and the VIF for all these predictor variables was less than 7.0, within the lower limit of values generally acknowledged to be problematic (5–10), suggesting little to no multicollinearity among the final predictor variables in our dataset (except between Urban cover and ALAN – discussed below in Section S4). This analysis resulted in a final predictor set of 8 variables for inclusion in the GAMs: maximum temperature, rainfall, ALAN, and land cover under arable, grassland, urban, and woodland categories.

S4. Generalized Additive Models (GAMs)

As previously mentioned, the arthropod abundance estimates correspond to volume densities within a specific height band. To model their spatio-temporal variation, we focused on estimates from a single, low-height band between 500 and 699 meters, which was represented in the maximum number of CVPs per radar (lower bands at 100 and 300 m were not available for all CVPs due to radar beam angle; Fig 1b). This decision was also based on the significant variation we observed in both, the arthropod abundance across heights (Fig. S4) and the number of height bands per CVP (Fig. 1b). Additionally, although we currently cannot segregate distinct taxonomic groups, it is reasonable to assume that different species or groups of species stratify across various height layers in the atmosphere based on several factors such as body size, dispersal ability and/or wing loading, and ecological characteristics (e.g., active migration or passive transport by thermal air currents).

Using GAMs, we evaluated the variation in estimated aerial arthropod abundance between 500 and 699 meters in height, considering spatial, temporal, and environmental variables. We compared seven alternative types of functional (smoothing) variations, $f(x)$, that can be fitted with GAMs to each, diurnal and nocturnal abundances. These models differed based on whether the smoothing function was allowed to vary individually for each radar and/or month, and whether the random effects of month and/or CVP grid location were included. The full model formulae for these different hierarchical GAMs are listed in Table S1, which also shows the differences in AIC and deviance explained (equivalent to Adj. R sqrd.) across them.

The final model (Table S1; top row) was selected using a combination of model diagnostics: normality and spread of the residuals (Fig. S6), k-index (values listed in Tables S3 & S5 for diurnal and nocturnal, respectively), deviance explained and ΔAIC (Table S1). This model included the random effects of month, CVP Grid location, and radar as random effects. We used ACF functions to account for temporal autocorrelation in the data and residuals. To determine the value of

autocorrelation coefficient *rho*, we ran a 'base' model (AR1) and obtained *rho* using the function *start_value_rho*() from R package *itsadug;*[Main Ref. 34]) (Fig. S7):

g(μ) = f1(year) + f2(year_f, 're') + f3(radar, 're') + f4(month, 're') + f5(CVP_location, 're')

To account for spatial-autocorrelation, we have included latitude and longitude as a smoothed, interaction term (i.e. *s(x,y)*). Residual spatial autocorrelation was evaluated using correlograms based on Moran's I[21], using CVP centroids as the spatial coordinates (Fig. S8).

The best fitting model included the following terms:

$$g(\mu) = f1(year) + f2(year_f, \Re) + f3(radar, \Re) + f4(Year, by = Radar) + \text{¿}$$

$$f5(month, \Re) + f6(CVP_{location}, \Re) + f7(x, y)$$

along with the following 9 covariates:

$$f8(Tmax) + f9(Rain) + f10(Wind) + f11(Arable) + \text{¿}$$

$$f12(Urban) + f13(Woodland) + f14(Grassland) + f15(ALAN) + f16(Elevation)$$

We observed that two covariates – ALAN and Urban land cover – were strongly correlated with each other ($R^2 = 0.87$; Fig. S5), which could affect model estimates. However, we included both variables in the final GAMs since we were interested in evaluating the difference in their effects across the diurnal and nocturnal arthropods. To account for the collinearity, we replaced ALAN by residual-ALAN (residuals from the linear regression of ALAN on urban land cover) in the GAMs, to evaluate the effect of ALAN independent of its relationship with Urban cover (Fig. 9). Residual-ALAN is the excess of ALAN after subtracting the fit to the ALAN and urban cover relationship and is thus independent of underlying Urban area effects. Substituting ALAN by residual-ALAN in the final GAM models did not change the results indicating a 'true' effect of ALAN on diurnal arthropods independent of urban land cover (Fig. S10).

We used the function *predict.gam()* to generate yearly spatial maps of aerial arthropod abundances per km^3 of atmosphere (Fig. 5). Since the predictions are dependent on the model's efficacy in capturing each of these relationships, they may differ from the actual observed (and modelled) patterns presented in Fig. 4. The predictions are also smoother and more generalized than the actual modelled relationships because they do not capture the specific deviations due to the omission of random group effects (e.g., specific radar stations or years). Therefore, though the predictions are useful in inferring macroscale processes, the generalizations may result in underestimation of local-scale variability in trends which should be interpreted with caution.

In the main text, Main Figure 3 shows the effects of individual predictors on arthropod abundance in our observed data (3A = Effect of Year, 3B to 3G = Effects of individual environmental covariates) – thus it shows the smoothing functions *f1, f8, f11, f12, f13*, and *f15* from the equation above and each smoothing function is calculated after accounting for the random effects of year factor, radar, month and CVP grid ID. Figure 4 shows the effect of only latitude and longitude in our observed data - i.e. it shows only the smoothing function f7 from the formula above – but again calculated after accounting for the random effects of year factor, radar, month and CVP grid ID. Figure 5, on the other hand, shows the cumulative effect of "all modelled variables" to predict abundances for unobserved data - i.e., it shows the cumulative effect of f1 through f16, but does not account for any random effects. Even for the coordinates included in Figure 4, Figure 5 will not show the exact same values in arthropod abundance – because predictions do not take random effects into account (more on this below). In Figure 4, the output thus reflects observed abundance patterns over space, as explained by the model, conditional on all the empirical data. In Figure 5, the random effects cannot be meaningfully assigned to new locations. Thus the entire predicted data (i.e., the spatial map) is a generalized prediction, not constrained by the empirical data directly although the empirical data was used for the 'training'.

S5. GAMs for individual height bands

We ran different GAMs for the height-specific estimates of aerial arthropod abundance. In the main text and elsewhere, we have discussed only the results from a single, low elevation band (500-699m). In this section we briefly cover the main results from the remaining models (which will be the focus of our future research). Overall, we found that the goodness of model fits at individual heights (measured as deviance explained, which is equivalent to Adjusted R-squared for Gaussian distributions) decreased with increasing heights. Complete model summaries for each individual height band, for diurnal and nocturnal datasets, are included as Dataset S1. The key observations have been summarized in Table S6, and are discussed below:

1. Maximum temperature (Tmax) was a consistently significant factor that appears to be a crucial determinant of abundance of arthropods in the air (in other words, aerial activity). Tmax generally exhibited a positive relationship with abundance during both diurnal and nocturnal periods. During the day, higher temperatures at different heights could be associated with increased thermal uplift, which may facilitate greater movement and activity of arthropods. At night, despite cooler overall conditions, temperature still plays a crucial role, possibly in maintaining metabolic rates and activity levels, or due to residual thermal updrafts from the ground.

2. Wind speed generally has a negative impact, particularly during nocturnal hours. This relationship is consistent across different heights, although its strength may vary. At lower heights, wind might limit the activity of smaller species more directly by increasing the energy costs of flight or movement. At higher heights, wind patterns could become more variable and less predictable, potentially leading to greater avoidance behaviours or altered migration routes. This highlights the complex role of wind, not only as a direct physical barrier but also as a determinant of energy expenditure and migratory patterns. We add caution that all the modelled relationships discussed here are derived from surface winds, which may not translate to the wind patterns at different heights. Therefore, we have currently refrained from an in-depth exploration of the implications of our findings, particularly in the context of how aerial dispersal mechanisms operate and how this might affect our understanding of arthropod migration patterns in high-altitude environments. We suggest that the inclusion of stratified environmental data, such as height-specific temperature, wind speed, and other variables, could enhance future models. This will be a focus in our future work.

3. Rainfall exhibited a relatively more variable effect depending on the time of day and height in the air column, suggesting that perhaps the direct influence of rain is more localized and other environmental conditions take precedence.

5. Artificial Light at Night (ALAN) is a significant factor predominantly during nocturnal periods, where it has a significant relationship with abundance across space. This effect could be attributed to the attraction of nocturnally active groups to light sources, which disrupt natural behaviour and potentially increase predation risks. However, the impact of ALAN decreases with height, and this could be attributed to the artificial light sources becoming less visible at higher heights, leading to a weaker influence on arthropod abundances and distributions.

6. Land cover variables—such as urban, arable, woodland, and grassland—decreased in significance at higher heights. This suggests that arthropod abundance at higher heights may be increasingly decoupled from ground-based environmental parameters. Possibly, the individuals at higher heights are involved in long-distance migration and therefore are affected by broader ecological processes acting at larger spatial scales than our CVP resolution (2.5km radius).

S6. Comparison with VLRs

Previous studies characterizing aerial arthropod activity and densities using remote sensing have primarily focused on Vertical Looking Radars (VLRs) and have revealed several key findings[22-30]. We discuss some of these below, which we were able to observe using dual-polarized data products from weather radars.

1. **migratory flight commences around midmorning and at dusk[23-25]:** Through high-resolution time-series profiling of radar differential reflectivity (ZDR), we observed diurnal and nocturnal peaks in arthropod activity (Fig. S2), which aligns with high aerial arthropod activity. Our inference, that these peaks in ZDR correspond to high aerial activity of arthropod targets, is based on the high correlation that we observed between ZDR and arthropod abundance in Rothamsted suction traps (Fig 2b). Although we chose broader time windows (0800-1400 hrs and 1800-2200 hrs) as compared to the narrower 'dawn' and 'dusk' periods of high activities reported in earlier studies[24,25], this was to accommodate the extensive latitudinal coverage of our data and standardize the sampling periods across this range.
2. **flights are mostly confined to the period between May and September, even though many species in the UK emerge as early as late April or as late as early October[26]** We observed strong, discernible peaks in ZDR between April and October across all 15 radars in the UK (Fig S2).
3. **Arthropods frequently reach altitudes between 100 and 1500 m, with occasional occurrences up to 2000 m[27]:** We obtained high density of arthropods up to 700 m and moderate densities up to at least 2100 m (Fig S4)
4. **A negative relationship exists between aerial arthropod density and altitude in the air column[27,28]:** We observed a strong non-linear negative relationship between arthropod density and height of the CVP band (Fig S4)

References

1. A.M. Dokter, P. Desmet, J.H. Spaaks, H.S. Van, L. Veen, L. Verlinden, C. Nilsson, G. Haase, H. Leijnse, A. Farnsworth, W. Bouten, J. Shamoun-Baranes, bioRad: biological analysis and visualization of weather radar data. *Ecography 42*, 852 – 860 (2019)
2. Pope, Addy. (2017). GB SRTM Digital Elevation Model (DEM) 90m, [Dataset]. EDINA. https://doi.org/10.7488/ds/1928.
3. J.R. Riley, Radar cross section of arthropods. Proceedings of the IEEE *73*, 228–232 (1985)
4. W. Li, C. Hu, R. Wang, S. Kong, F. Zhang, Comprehensive analysis of polarimetric radar cross-section parameters for arthropod body width and length estimation. *Science China Information Sciences 64*, e122302 (2021)
5. R. Wang, X. Kou, K. Cui, H. Mao, S. Wang, Z. Sun, W. Li, Y. Li, C. Hu, Arthropod-Equivalent Radar Cross-Section Model Based on Field Experimental Results of Body Length and Orientation Extraction. *Remote Sensing 14*, e508 (2022)
6. S. Kong, C. Hu, R. Wang, F. Zhang, L. Wang, T. Long, K. Wu, Arthropod multifrequency polarimetric radar cross section: Experimental results and analysis. *IEEE Transactions on Geoscience and Remote Sensing 59*, 6573 – 6585 (2020)
7. Drake, V.A.; Chapman, J.W.; Lim, K.S.; Reynolds, D.R.; Riley, J.R.; Smith, A.D. Ventral-aspect radar cross sections and polarization patterns of insects at X band and their relation to size and form. *Int. J. Remote Sens.* 2017, *38*, 5022–5044, doi:10.1080/01431161.2017.1320453.
8. Thurai, M., D. Hudak, and V. N. Bringi. "On the possible use of copolar correlation coefficient for improving the drop size distribution estimates at C band." *Journal of Atmospheric and Oceanic Technology* 25, no. 10 (2008): 1873-1880.
9. Yan DI, Qi-lin WA, Ling YA, Xian-tong LI, Feng XI, Lu FE. Raindrop size distribution parameters retrieved from xinfeng c-band polarimetric radar observations. Journal of Tropical Meteorology. 2020 Sep 1;26(3):275-85.
10. Met Office; Hollis, D.; McCarthy, M.; Kendon, M.; Legg, T.; Simpson, I. (2018): HadUK-Grid gridded and regional average climate observations for the UK. Centre for Environmental Data Analysis, *2023-08-02 http://catalogue.ceda.ac.uk/uuid/4dc8450d889a491ebb20e724debe2dfb/*
11. X. Li, Y. Zhou, M. Zhao, X. Zhao, A harmonized global nighttime light dataset 1992–2018. *Scientific data 7*, e168 (2020)
12. Land Cover Map 2021 (10m classified pixels, GB): Marston, C.; Rowland, C.S.; O'Neil, A.W.; Morton, R.D. (2022). Land Cover Map 2021 (10m classified pixels, GB). NERC EDS Environmental Information Data Centre. https://doi.org/10.5285/a22baa7c-5809-4a02-87e0-3cf87d4e223a
13. DOI for Land Cover Map 2020 (10m classified pixels, GB): Morton, R.D.; Marston, C.G.; O'Neil, A.W.; Rowland, C.S. (2021). Land Cover Map 2020 (10m Classified Pixels, GB). NERC EDS Environmental Information Data Centre. https://doi.org/10.5285/35c7d0e5-1121-4381-9940-75f7673c98f7

14. DOI for Land Cover Map 2019 (25m rasterised land parcels, GB): Morton, D.; Marston, C. G, O'Neil, A. W.; Rowland, C. S. (2020). Land Cover Map 2019 (25m rasterised land parcels, GB). NERC Environmental Information Data Centre. https://doi.org/10.5285/f15289da-6424-4a5e-bd92- 48c4d9c830cc
15. DOI for Land Cover Map 2018 (20m classified pixels, GB): Morton, R.D.; Marston, C.G.; O'Neil, A.W.; Rowland, C.S. (2020). Land Cover Map 2018 (20m classified pixels, GB). NERC Environmental Information Data Centre. https://doi.org/10.5285/b3dfc4c7-c9bd-4a02-bed8-46b2a41be04a
16. DOI for Land Cover Map 2017 (20m classified pixels, GB): Morton, R.D; Marston, C.G.; O'Neil, A.W; Rowland, C.S. (2020). Land Cover Map 2017 (20m classified pixels, GB). NERC Environmental Information Data Centre. https://doi.org/10.5285/f6f86b1a-af6d-4ed8-85af-21ee97ec5333
17. Rowland, C.S.; Morton, R.D.; Carrasco, L.; McShane, G.; O'Neil, A.W.; Wood, C.M. (2017) Land Cover Map 2015 (25m raster, GB). NERC Environmental Information Data Centre. https://doi.org/10.5285/bb15e200-9349-403c-bda9-b430093807c7
18. Morton, R.D.; Rowland, C.S.; Wood, C.M.; Meek, L.; Marston, C.G.; Smith, G.M. (2014). Land Cover Map 2007 (25m raster, GB) v1.2. NERC Environmental Information Data Centre. https://doi.org/10.5285/a1f88807-4826-44bc-994d-a902da5119c2
19. Fuller, R.M.; Smith, G.M.; Sanderson J.M.; Hill, R.A.; Thomson, A.G; Cox, R.; Brown, N.J.; Clarke, R.T; Rothery, P.; Gerard, F.F. (2002). Land Cover Map 2000 (25m raster, GB). NERC Environmental Information Data Centre. https://doi.org/10.5285/f802edfc-86b7-4ab9-b8fa-87e9135237c9
20. S. Moritz, B. Thomas, imputeTS: time series missing value imputation in R. *The R Journal 9*, e207 (2017)
21. Moran, Patrick AP. "A test for the serial independence of residuals." *Biometrika* 37, no. 1/2 (1950): 178-181.
22. J.W. Chapman, V. Drake, D.R. Reynolds, Recent Insights from Radar Studies of Arthropod Flight. *Annual Review of Entomology 56*, 337 – 356 (2011)
23. Reid, D. G., Wardhaugh, K. G., & Roffey, J. (1981). Radar studies of insect flight at Benalla, Victoria, in February 1974.
24. Drake, V. A., & Farrow, R. A. (1983). The nocturnal migration of the Australian plague locust, Chortoicetes terminifera (Walker)(Orthoptera: Acrididae): quantitative radar observations of a series of northward flights. *Bulletin of Entomological Research*, *73*(4), 567-585.
25. Riley, J. R., & Reynolds, D. R. (1979). Radar-based studies of the migratory flight of grasshoppers in the middle Niger area of Mali. *Proceedings of the Royal Society of London. Series B. Biological Sciences*, *204*(1154), 67-82.
26. Hu, G., Lim, K. S., Horvitz, N., Clark, S. J., Reynolds, D. R., Sapir, N., & Chapman, J. W. (2016). Mass seasonal bioflows of high-flying insect migrants. *Science*, *354*(6319), 1584-1587.

27. V.A. Drake, the vertical distribution of macro-arthropods migrating in the nocturnal boundary layer: A radar study. Boundary-Layer Meteorology *28*, 353 – 374 (1984)
28. C.G. Johnson, The distribution of arthropods in the air and the empirical relation of density to height. *The Journal of Animal Ecology 1957*, 479 – 494 (1957)
29. J.W. Chapman, D. Reynolds, A.D. Smith, J.R. Riley, D.E. Pedgley, I.P. Woiwod, High-altitude migration of the diamondback moth *Plutella xylostella* to the U.K.: a study using radar, aerial netting, and ground trapping. *Ecological Entomology 27*, 641 – 650 (2002)
30. C.R Wood, J. Chapman, D.R Reynolds, J.F. Barlow, A.D. Smith, I.P. Woiwod, The influence of the atmospheric boundary layer on nocturnal layers of noctuids and other moths migrating over southern Britain. *International Journal of Biometeorology 50*, 193 – 204 (2006)

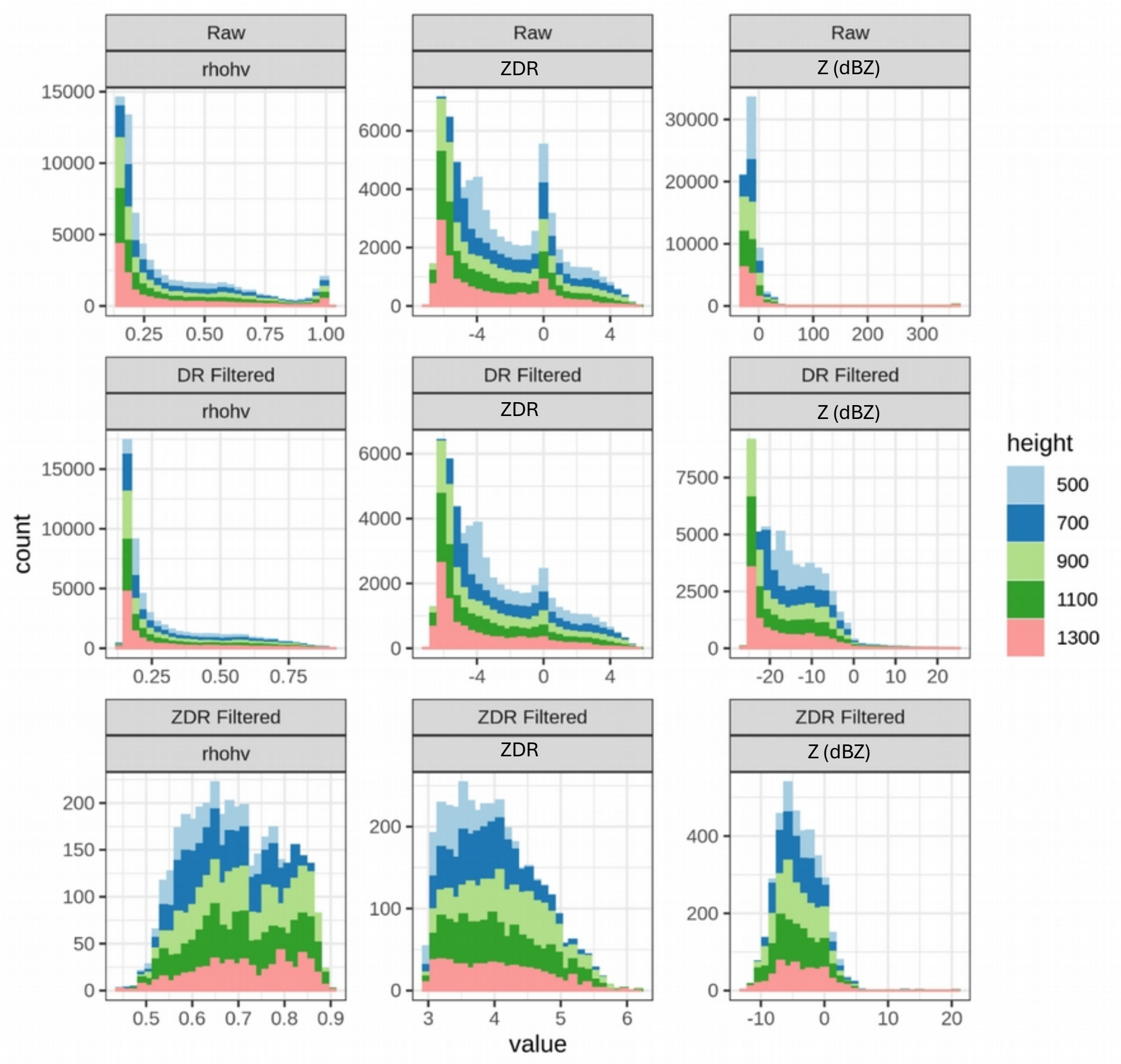

Figure S1. Distribution of three radar variables across the raw (top row) and processed (DR Filtered; middle row, and ZDR Filtered; bottom row) data. We removed all meteorological signals that could be attributed to precipitation using the 'DR-Filtering' method developed by Kilambi et al.Main Ref. No. 26. A depolarization ratio (DR) was calculated using polarimetric variables ZDR and ρHV, and all data below a DR threshold of −12.5 dB was attributed to precipitation and removed. We also removed all data with extremely high reflectivity (>45 dBZ) which are often associated with heavy rainfall but may not be efficiently captured by the depolarization ratio. On this filtered data, a ZDR threshold of +3 dB was further applied to further increase the signal to noise ratio of (elongated) arthropods. The data shown above corresponds to a random subset of 100 days from the Chenies radar and is shown to emphasize that the final processed (filtered) dataset used for arthropod abundance estimation (bottom row) corresponds to a fraction of the volume of the original meteorological datasets (top row).

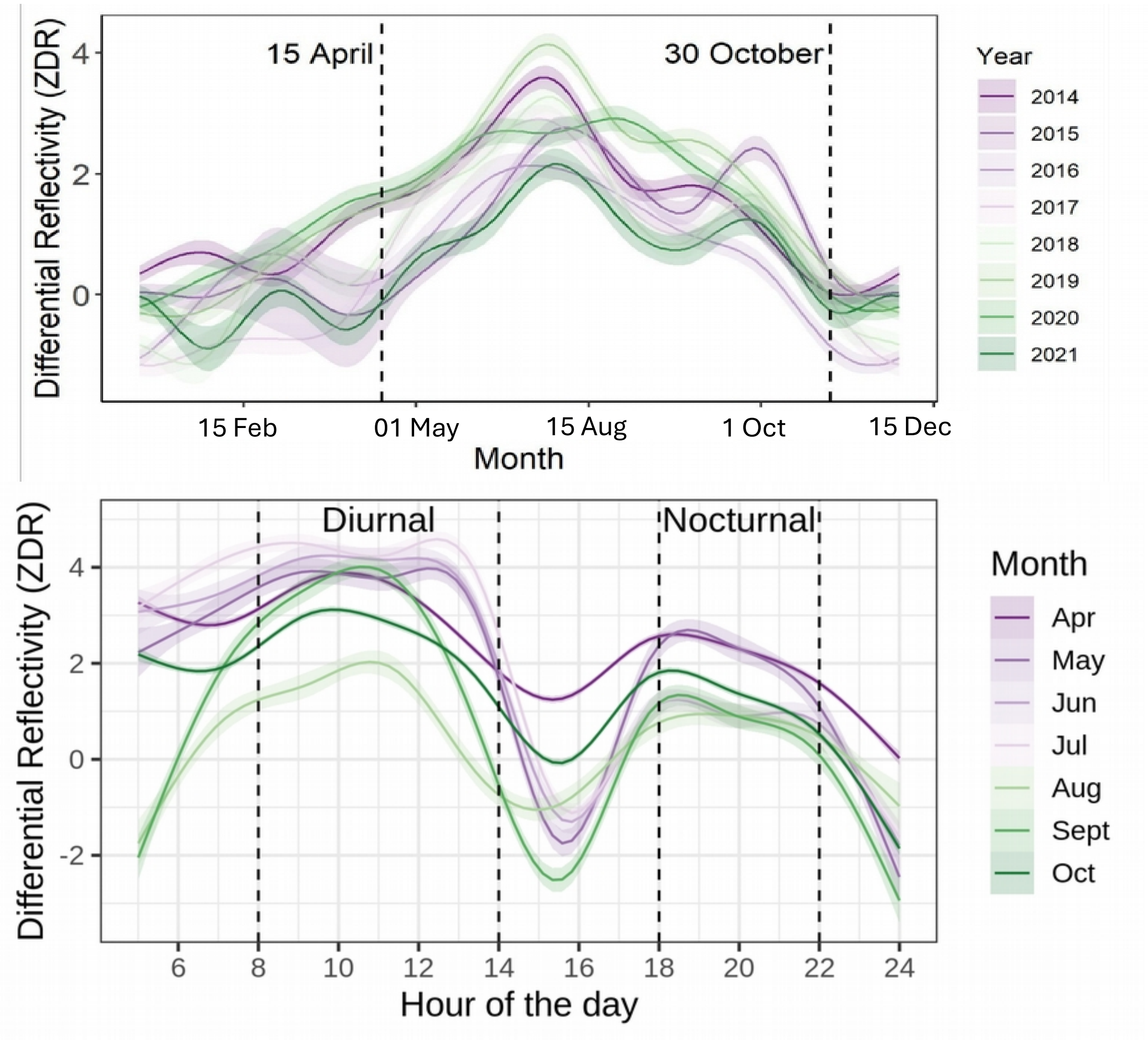

Figure S2. Generalized Additive Model (GAM) curves fit to the time series profiles of Radar differential reflectivity (ZDR). Raw ZDR is a measure of the log of the ratio of the horizontal reflectivity to vertical reflectivity; ZDR = $10\log_{10}(\eta_h/\eta_v)$, where η is the reflectivity. When horizontal and vertical reflectivity are identical, i.e. the target is a perfect reflecting sphere, ZDR = 0; for a horizontally oriented target (e.g. elongated arthropods) ZDR > 0. ZDR values from across the 15 UKMO radars were modelled using Generalized Additive Models (GAMs), which was used to identify the peaks in ZDR, characteristic of high arthropod activity in the air (also see Fig. S6). Based on cubic spline fitted to each year separately, we use a window of 15th April to 30th October for truncating the Radar data in all downstream analyses. Using diurnal data, we further restricted our analysis to a single scan per two distinct time windows: diurnal (maximum ZDR between 0800 to 1400 GMT), and nocturnal (maximum ZDR between 1800 to 2200 GMT) to estimate the abundance of aerial arthropods.

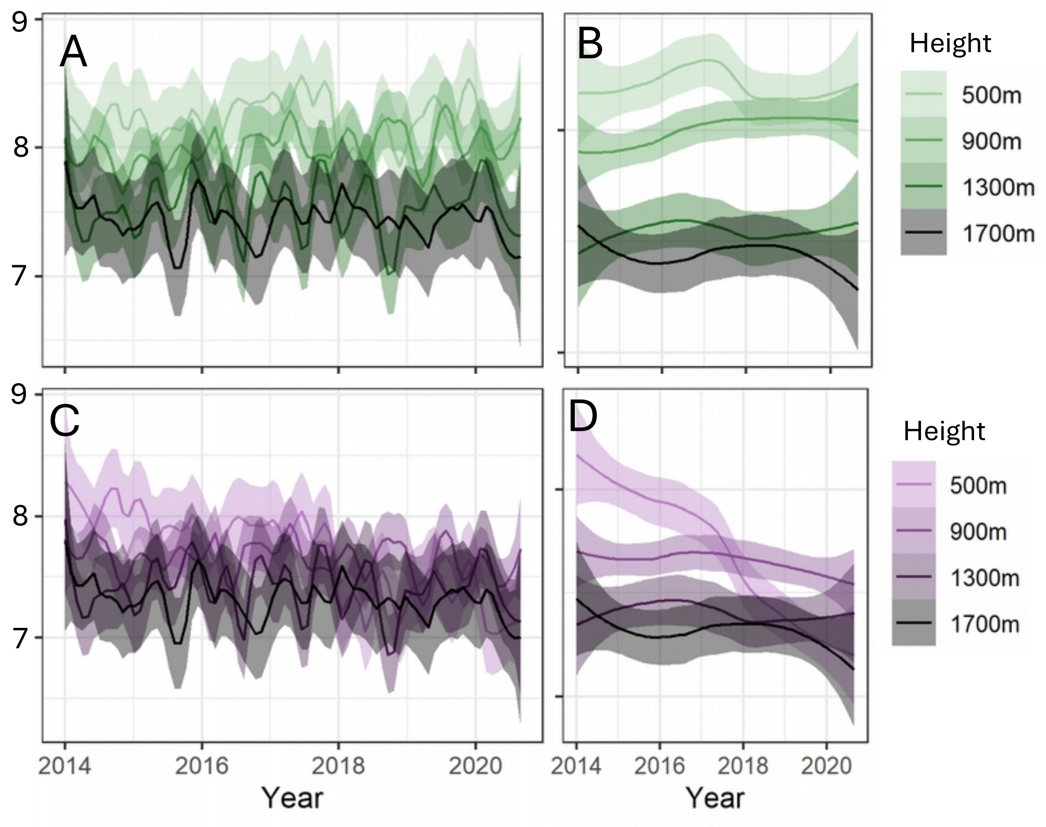

Figure S3. Raw temporal trends of cumulative aerial abundance of diurnal (in green; top row) and nocturnal (in purple; bottom row) arthropods estimated from 15 weather radars across the United Kingdom. A) and B) show the raw (daily) and smoothed (aggregated for each month) temporal trends, respectively for diurnal aerial arthropods estimated at different heights in the air column using reflectivity data from weather radars. C) and D) show similar profiles for nocturnal aerial arthropods. The heights noted in the legend correspond to the lower limit for each corresponding 200 m band within which arthropod abundances are calculated – i.e. 500m corresponds to a columnar band between 500 – 700m; 900m = 900 – 1100m; and so on. For clarity, only 4 bands are shown here, even though the abundances are calculated for 9 bands between 100m and 2100m. Fitted lines are loess smooths, and do not account for the variation in data from across different radars

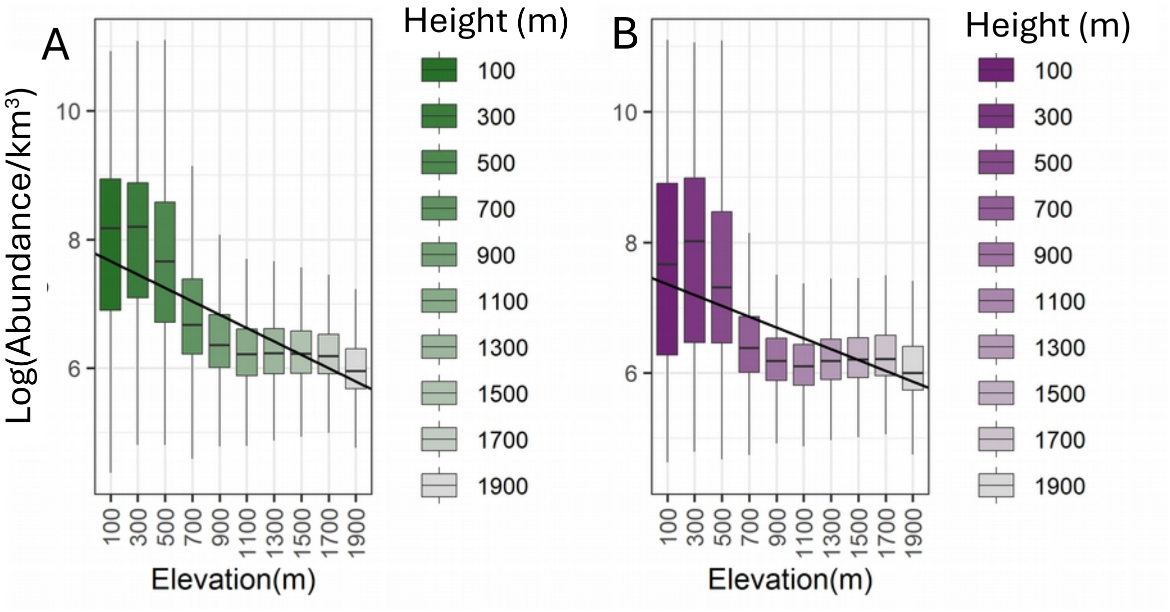

Figure S4. Relationship between estimated aerial diurnal and nocturnal arthropod abundances and height in the air column. Ordinary least square regression was fitted to the relationship between estimated arthropod abundances (volume density inside each height band of a CVP) and the band heights. Log abundances are shown on the y-axis to account for the huge variation in values across different heights. Converting to linear scales, on average, arthropod abundances decreased monotonically at the rate of 8.74 (±0.01) x 10^5 individuals per 200 m of height gained in the air column. Slopes on linear scales: Diurnal = (-7.77 ± 0.01) x 10^5, Adj. R^2 = 0.11, $p < 0.001$; and Nocturnal = (-9.71 ± 0.21) x 10^5, Adj. R^2 = 0.12, $p < 0.001$).

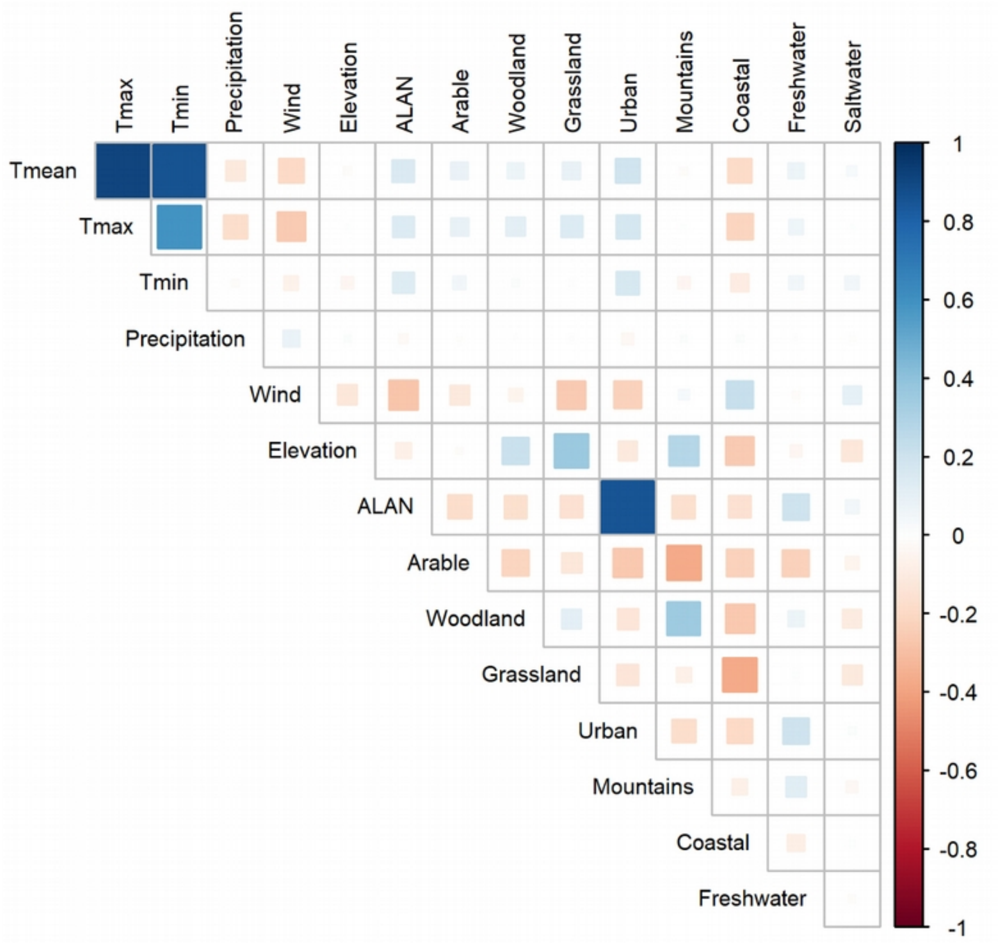

Figure S5. Pairwise Pearson's correlation coefficients showing the multi-collinearity between different environmental and landscape variables used in the present study. We assembled 15 environmental variables at the CVP spatial resolution, known to influence arthropod populations: surface mean, minimum and maximum daily temperatures (TMean, TMin, and TMax respectively), daily precipitation, daily average surface wind speeds, Elevation (above mean sea level; obtained from a 90m Digital Terrain Model), Artificial Lights At Night (ALAN; Li et al. 2018), and percentage land cover under arable, woodland, grassland, urban, mountains, coastal, saltwater and freshwater. To assess multicollinearity between the different environmental variables, we used Pearson's correlation coefficients and removed variables which were strongly correlated with one or more of the remaining, resulting in a final predictor set of 8 variables for inclusion in the GAMs: maximum temperature, rainfall, ALAN, and land cover under arable, grassland, urban, and woodland categories.

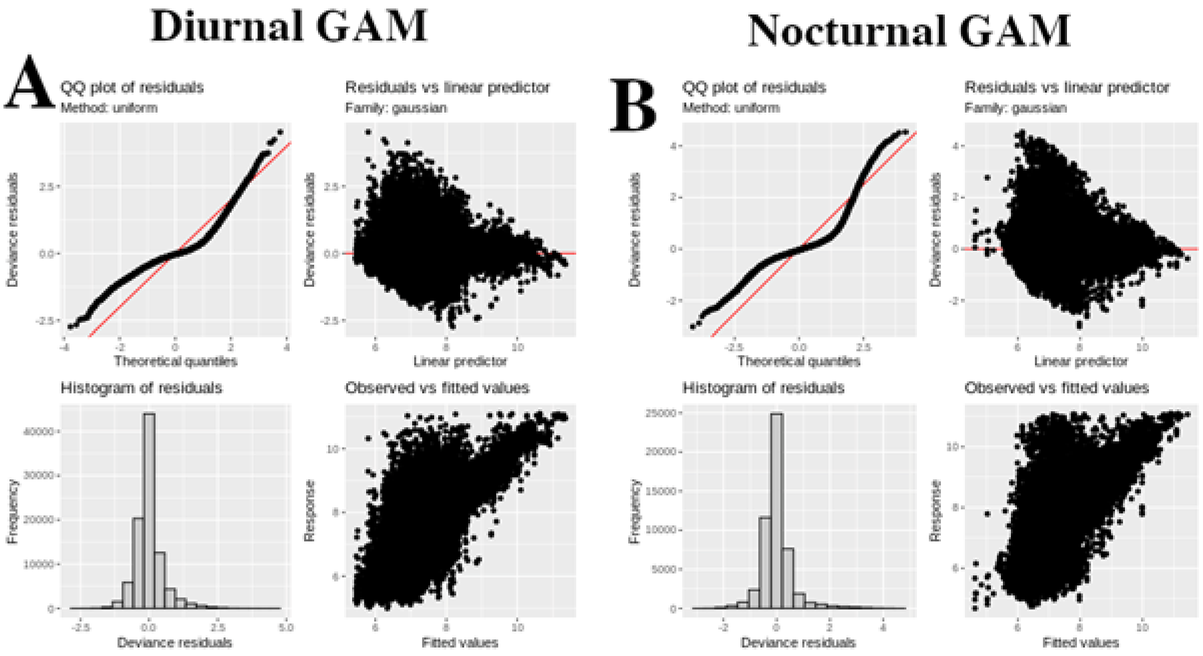

Figure S6. Diagnostic plots for Generalized Additive Models fitted to the 8 year (2014 - 2021) diurnal and nocturnal aerial arthropod abundance data from across 15 Radars in the United Kingdom. Each figure comprises four plots: QQ plot of residuals, residual versus predictor, normality of residuals, and correlation between observed values and fitted model.

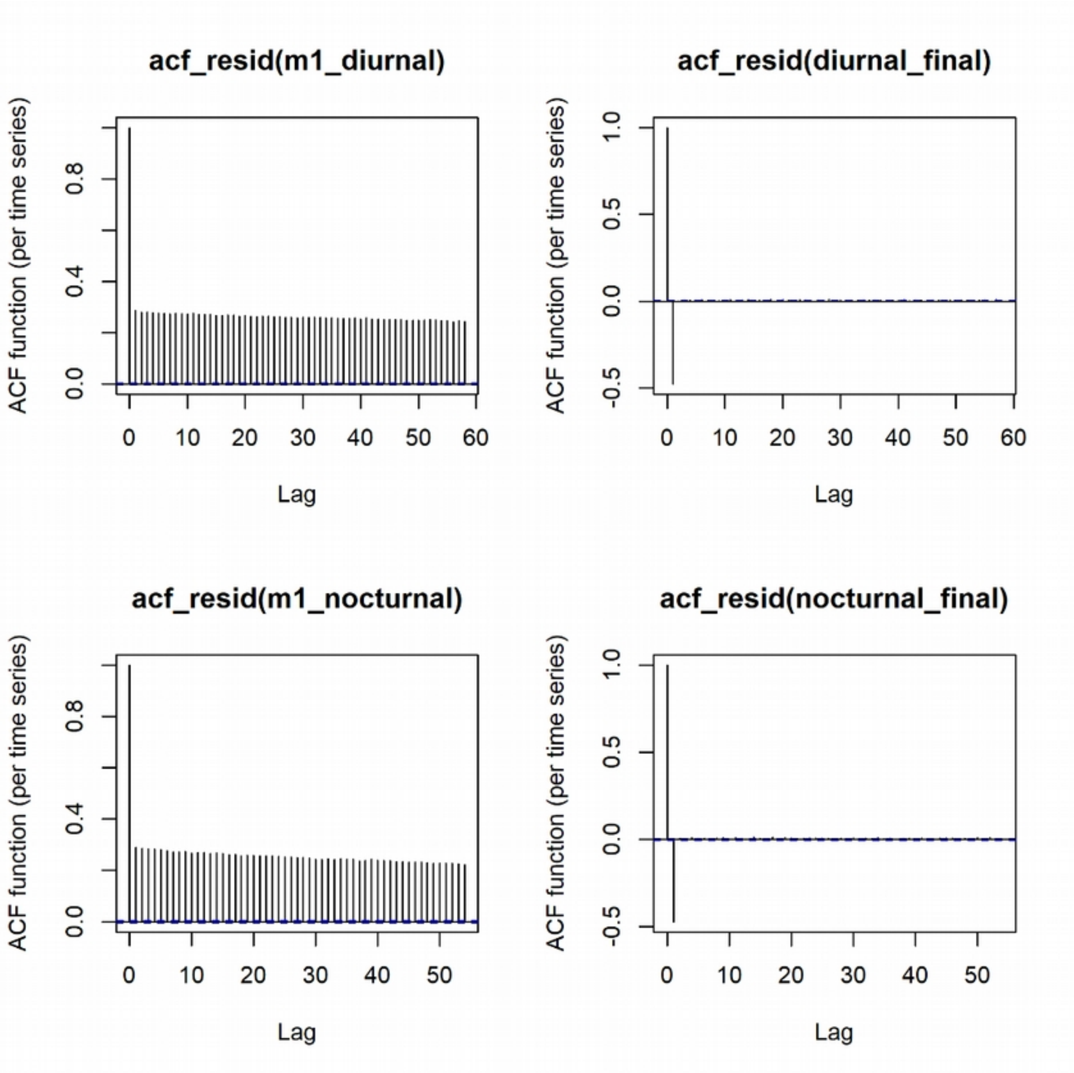

Figure S7. Autocorrelation function (ACF) plot of generalised additive model residuals from a base model assuming independent residuals (left), compared with a model corrected with autoregression (right) for spatio-temporal modelling of diurnal (top row) and nocturnal (bottom row) aerial arthropod abundances. Left panels show the autocorrelation function (ACF) plot of model residuals for the "base model" determining the value for the autocorrelation coefficient 'rho':

$$g(\mu) = f1(year) + f2(yearf,'re') + f3(radar,'re') + f4(month,'re') + f5(CVPlocation,'re')$$

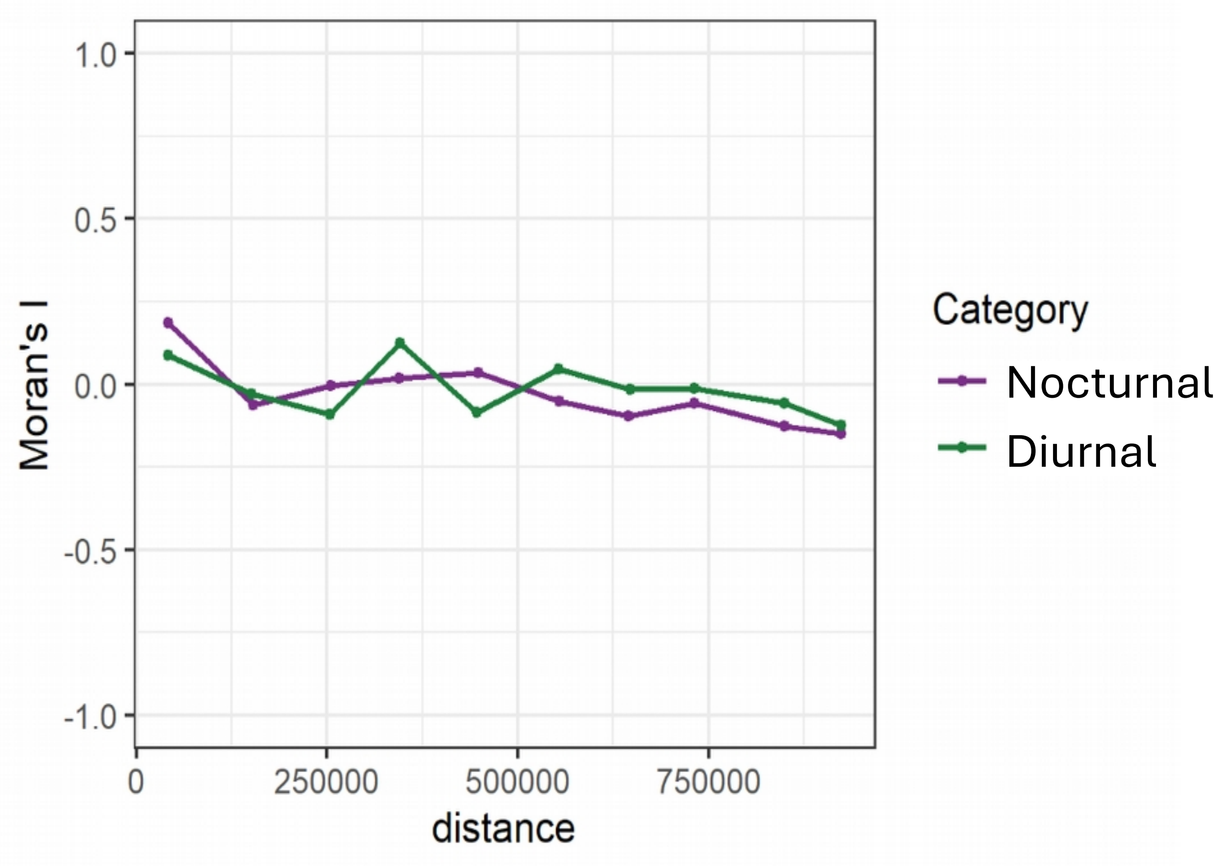

Figure S8. Moran's correlogram for the residuals from two Generalized Additive Models for diurnal (purple) and nocturnal (green) aerial arthropod abundances

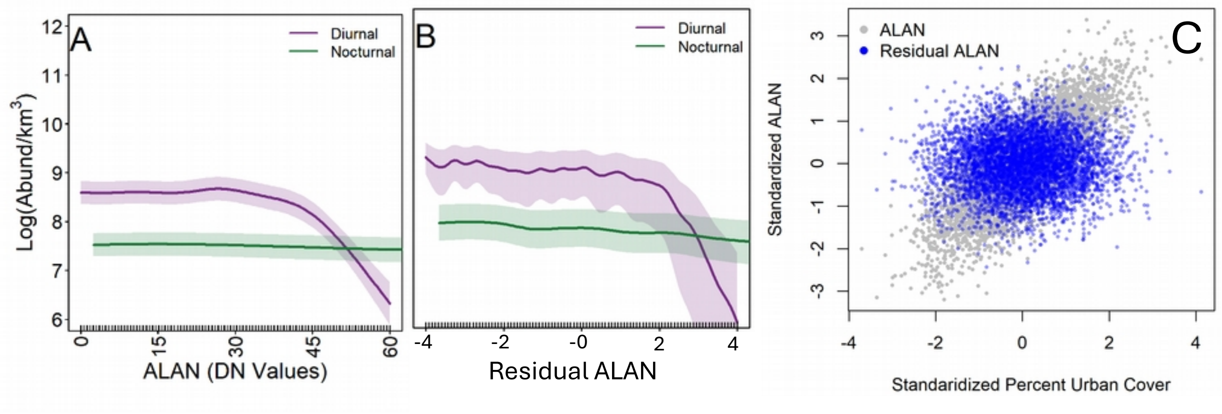

Figure S9. Accounting for the ALAN-Urban cover collinearity. A) Shows the effect of artificial light at night (ALAN) on log-abundance of aerial arthropods, when another strongly correlated variable (% Urban land cover) is also included in the model. To evaluate the independent effect of the two covariates – ALAN and Urban land cover – which were strongly correlated with each other, we replaced ALAN by residual-ALAN in the GAMs, to evaluate the effect of ALAN independent of its relationship with Urban cover (shown in **B**). **C** shows the linear regression of the two variables (ALAN; y-axis) and Urban land cover (x-axis) shown in grey colour. Residual ALAN (blue) was obtained by differencing the linear coefficients of this fit from ALAN.

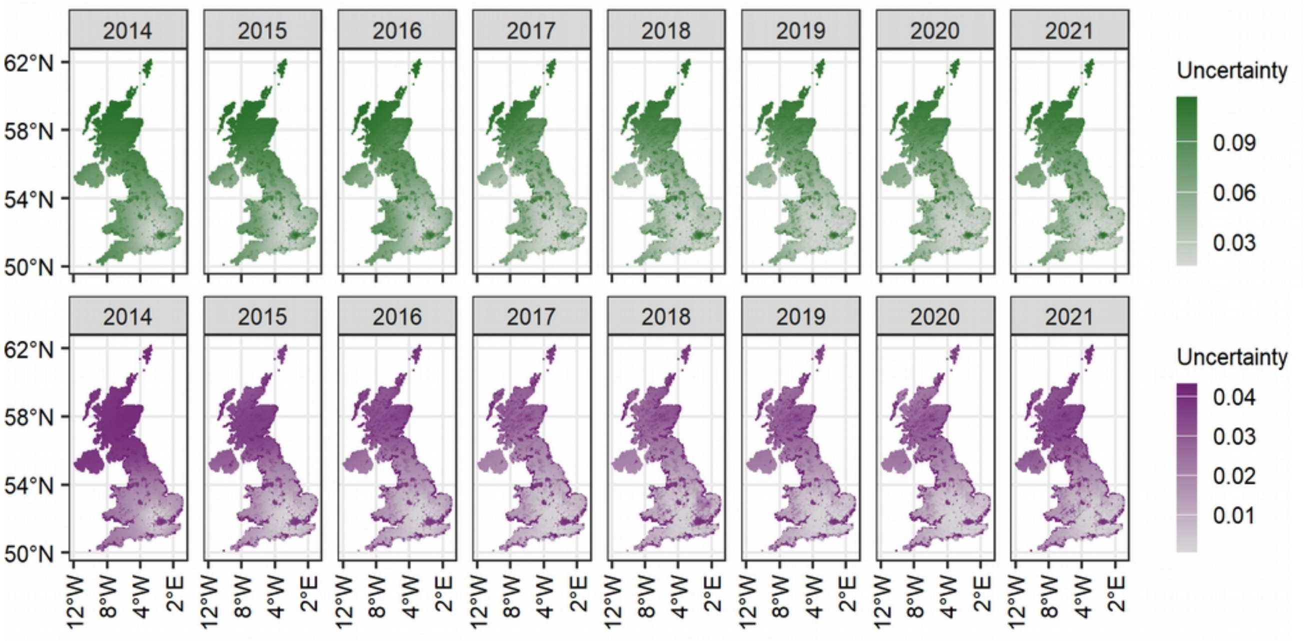

Figure S10. The uncertainty (standard errors) of spatio-temporal Generalized Additive Model (GAM) fitted to diurnal (top)- and nocturnal (bottom) aerial arthropod abundances across the UK. The predictions are derived from the spatio-temporal patterns of underlying covariates such as weather, land cover, elevation, and artificial light at night (ALAN), combined with the modelled relationship between these covariates and arthropod abundance. The predictions are based only on the fixed effects, which represent general relationships between covariates (e.g., weather, land cover) and the response variable (arthropod abundance), and excludes the random effects, thereby making the predictions smoother and more generalized. The individual temporal raster of each of predictor variable used in GAM were stacked together for predictions. The higher standard errors associated with coastal, and a few urban cities may be attributed to (i) the absence of coastal data from our models, and (ii) high fluctuations in daily arthropod abundance over the cities. We note that the model uncertainties, despite high spatial variation, are relatively low across all sites.

Table S1. Full Model formulae for the different hierarchical GAMs for modeling the spatio-temporal variation in aerial arthropod abundances. There were 9 covariates included in all the models listed along with four interaction terms (not included in the model formulae shown below. Each new term added to the base model (bottom row) is highlighted in blue. ΔAIC is the difference between pairwise models. Dev. Exp. (%) is the percentage deviance explained by the respective model and is equal to 2logL(saturated model | data) − 2logL(model | data).

	**Diurnal**		**Nocturnal**	
	**Dev. Exp. (%)**	**ΔAIC**	**Dev. Exp. (%)**	**ΔAIC**
y ~ s(Year) + ti(x, y, Year) + s(Year_factor, bs = "re") + s(Radar, bs = "re") + **s(Year, by = Radar) + s(Month, bs = “re”) + s(Grid_ID, bs = “re”) + s(x,y)**	80.2	0	76.4	0
y ~ s(Year) + ti(x, y, Year) + s(Year_factor, bs = "re") + s(Radar, bs = "re") + **s(Year, by = Radar) + s(Month, bs = “re”) + s(Grid_ID, bs = “re”)**	80.3	-21	76.7	-13
y ~ s(Year) + ti(x, y, Year) + s(Year_factor, bs = "re") + s(Radar, bs = "re") + **s(Year, by = Radar) + s(Month, bs = “re”) + s(Grid_ID, bs = “re”) + s(Month, Radar)**	46.4	-4800.1	27.8	-1081.1
y ~ s(Year) + ti(x, y, Year) + s(Year_factor, bs = "re") + s(Radar, bs = "re") + **s(Year, by = Radar) + s(Month, bs = “re”) + s(Grid_ID, bs = “re”)**	46.7	-2986.3	23.1	-913.1
y ~ s(Year) + ti(x, y, Year) + s(Year_factor, bs = "re") + s(Radar, bs = "re") + **s(Year, by = Radar) + s(Month, bs = “re”)**	43.4	-6894.3	23.2	-920.2
y ~ s(Year) + ti(x, y, Year) + s(Year_factor, bs = "re") + s(Radar, bs = "re") + **s(Year, by = Radar)**	40.2	-7428.5	21.2	-1290.5
y ~ s(Year) + ti(x, y, Year) + s(Year_factor, bs = "re") + s(Radar, bs = "re")	38.5	-8285.6	17.8	-1366.6

Table S2. Full model summary, including significance of covariates and different terms, for the final GAM fitted to the 8-year (2014 - 2021) diurnal aerial arthropod abundance data from across 15 Radars in the United Kingdom.

term	edf	ref.df	statistic	p.value
s(Year)	0	5	0.00	0.78
s(Year_factor)	4	8	47.18	0.00
s(Radar)	11	14	23.58	0.00
s(Year):RadarCastor-Bay	0	5	0.00	0.35
s(Year):RadarChenies	3	5	49.28	0.00
s(Year):RadarCobbacombe-Cross	2	4	3.94	0.00
s(Year):RadarCrug-y-Gorllwyn	0	4	0.00	0.73
s(Year):RadarDean-Hill	0	5	0.00	0.65
s(Year):RadarDruim-a-Starraig	0	4	0.00	0.54
s(Year):RadarHameldon-Hill	4	5	23.96	0.00
s(Year):RadarHigh-Moorsley	3	5	26.41	0.00
s(Year):RadarHill-of-Dudwick	4	5	46.96	0.00
s(Year):RadarHolehead	0	4	0.00	0.26
s(Year):RadarIngham	0	4	0.00	0.41
s(Year):RadarMunduff-Hill	3	4	16.23	0.00
s(Year):RadarPredannack	2	5	0.85	0.04
s(Year):RadarThurnham	3	5	22.24	0.00
s(Month)	6	7	63.46	0.00
s(x,y)	83	99	149.57	0.00
ti(Year,x,y)	73	96	19.42	0.00
s(ID)	94	131	4.26	0.00
s(Tmax)	21	29	27.52	0.00
s(Rain)	16	29	11.80	0.00
s(Wind)	14	29	4.49	0.00
s(Arable)	24	29	15.75	0.00
s(Urban)	20	29	10.87	0.00
s(ALAN)	25	29	12.00	0.00
s(Elevation)	2	2	101.37	0.00
s(Woodland)	13	29	9.35	0.00
s(Grassland)	22	29	19.89	0.00
ti(Tmax,Rain)	13	16	36.93	0.00
ti(Arable,ALAN)	11	16	4.46	0.00
ti(Tmax,ALAN)	10	16	10.30	0.00
ti(Woodland,Tmax)	10	16	5.09	0.00

Table S3. Results for the diagnostic tests of whether the basis dimension (k) choices are adequate for the final GAM fitted to the 8 year (2014 - 2021) diurnal aerial arthropod abundance data from across 15 Radars in the United Kingdom. Values of k-index closer to 0 indicate that the estimated degrees of freedom (edf) are very close to the available degrees of freedom (k` ), indicating that there is missed pattern left in the residuals. Low p-values may indicate that the basis dimension, k` , has been set too low, especially if the reported edf is close to k` . (Smooths of factor variables are not supported).

	k'	edf	k-index	p-value
s(Year)	5	0	0.99	0.27
s(Year_factor)	8	4	NA	NA
s(Radar)	14	11	NA	NA
s(Year):RadarCastor-Bay	5	0	0.99	0.25
s(Year):RadarChenies	5	3	0.99	0.24
s(Year):RadarCobbacombe-Cross	5	2	0.99	0.27
s(Year):RadarCrug-y-Gorllwyn	5	0	0.99	0.27
s(Year):RadarDean-Hill	5	0	0.99	0.24
s(Year):RadarDruim-a-Starraig	5	0	0.99	0.27
s(Year):RadarHameldon-Hill	5	4	0.99	0.20
s(Year):RadarHigh-Moorsley	5	3	0.99	0.22
s(Year):RadarHill-of-Dudwick	5	4	0.99	0.23
s(Year):RadarHolehead	5	0	0.99	0.20
s(Year):RadarIngham	5	0	0.99	0.26
s(Year):RadarMunduff-Hill	5	3	0.99	0.27
s(Year):RadarPredannack	5	2	0.99	0.23
s(Year):RadarThurnham	5	3	0.99	0.21
s(Month_abb)	7	6	NA	NA
s(x,y)	99	83	0.74	0.00
ti(Year,x,y)	96	73	0.79	0.00
s(ID)	131	94	NA	NA
s(Tmax)	29	21	0.97	0.02
s(Rain)	29	16	0.97	0.02
s(Wind)	29	14	0.88	0.00
s(Arable)	29	24	0.85	0.00
s(Built.up.gardens)	29	20	0.84	0.00
s(ALAN)	29	25	1.01	0.75
s(Elevation)	2	2	0.79	0.00
s(Woodland)	29	13	0.83	0.00
s(Grassland)	29	22	0.82	0.00
ti(Tmax,Rain)	16	13	1.01	0.78
ti(Arable,ALAN)	16	11	0.80	0.00
ti(Tmax,ALAN)	16	10	0.96	0.00
ti(Woodland,Tmax)	16	10	0.96	0.00

Table S4. Full model summary, including significance of covariates and different terms, for the final GAM fitted to the 8 year (2014 - 2021) nocturnal aerial arthropod abundance data from across 15 Radars in the United Kingdom.

term	edf	ref.df	statistic	p.value
s(Year)	0	5	0.00	0.00
s(Year_factor)	3	7	13.13	0.00
s(Radar)	11	13	11.79	0.00
s(Year):RadarCastor-Bay	1	5	0.59	0.03
s(Year):RadarChenies	0	5	0.00	0.39
s(Year):RadarCobbacombe-Cross	2	4	2.11	0.00
s(Year):RadarCrug-y-Gorllwyn	2	4	3.58	0.00
s(Year):RadarDean-Hill	0	5	0.00	0.66
s(Year):RadarDruim-a-Starraig	2	4	5.70	0.00
s(Year):RadarHameldon-Hill	0	5	0.00	0.31
s(Year):RadarHigh-Moorsley	2	5	1.14	0.01
s(Year):RadarHill-of-Dudwick	4	5	30.69	0.00
s(Year):RadarHolehead	3	4	9.91	0.00
s(Year):RadarIngham	1	4	1.62	0.00
s(Year):RadarMunduff-Hill	2	4	1.20	0.02
s(Year):RadarPredannack	3	5	5.05	0.00
s(Year):RadarThurnham	3	5	58.62	0.00
s(Month_abb)	6	6	63.66	0.00
s(x,y)	71	99	2649.33	0.00
ti(Year,x,y)	64	96	12.74	0.00
s(ID)	85	129	2.86	0.00
s(Tmax)	20	29	14.41	0.00
s(Rain)	11	29	12.40	0.00
s(Wind)	4	29	0.56	0.08
s(Arable)	16	29	3.78	0.00
s(Urban)	1	29	0.09	0.01
s(ALAN)	25	29	9.99	0.00
s(Elevation)	2	2	189.22	0.00
s(Woodland)	16	29	4.58	0.00
s(Grassland)	25	29	11.92	0.00
ti(Tmax,Rain)	14	16	22.60	0.00
ti(Arable,ALAN)	9	16	4.19	0.00
ti(Tmax,ALAN)	12	16	4.27	0.00
ti(Woodland,Tmax)	9	16	3.91	0.00

Table S5. Results for the diagnostic tests of whether the basis dimension choices are adequate for the final GAM fitted to the 8 year (2014 - 2021) nocturnal aerial arthropod abundance data from across 15 Radars in the United Kingdom. Values of k-index closer to 0 indicate that the estimated degrees of freedom (edf) are very close to the available degrees of freedom (k`), indicating that there is missed pattern left in the residuals. Low p-values may indicate that the basis dimension, **k`**, has been set too low, especially if the reported edf is close to k`. (Smooths of factor variables are not supported).

term	k'	edf	k-index	p-value
s(Year)	5	0.00	0.98	0.12
s(Year_factor)	8	3.27	NA	NA
s(Radar)	14	10.75	NA	NA
s(Year):RadarCastor-Bay	5	1.43	0.98	0.16
s(Year):RadarChenies	5	0.00	0.98	0.12
s(Year):RadarCobbacombe-Cross	5	1.99	0.98	0.10
s(Year):RadarCrug-y-Gorllwyn	5	1.93	0.98	0.14
s(Year):RadarDean-Hill	5	0.00	0.98	0.13
s(Year):RadarDruim-a-Starraig	5	2.41	0.98	0.11
s(Year):RadarHameldon-Hill	5	0.00	0.98	0.13
s(Year):RadarHigh-Moorsley	5	1.89	0.98	0.13
s(Year):RadarHill-of-Dudwick	5	3.93	0.98	0.15
s(Year):RadarHolehead	5	3.07	0.98	0.13
s(Year):RadarIngham	5	1.28	0.98	0.12
s(Year):RadarMunduff-Hill	5	1.55	0.98	0.10
s(Year):RadarPredannack	5	2.76	0.98	0.12
s(Year):RadarThurnham	5	3.25	0.98	0.13
s(Month_abb)	7	5.70	NA	NA
s(x,y)	99	71.27	0.71	0.00
ti(Year,x,y)	96	63.55	0.79	0.00
s(ID)	84	84.91	NA	NA
s(Tmax)	29	19.77	0.96	0.00
s(Rain)	29	11.27	0.97	0.02
s(Wind)	29	3.77	0.88	0.00
s(Arable)	29	16.14	0.82	0.00
s(Built.up.gardens)	29	0.60	0.8	0.00
s(ALAN)	29	24.83	0.97	0.01
s(Elevation)	2	1.77	0.77	0.00
s(Woodland)	29	16.23	0.81	0.00
s(Grassland)	29	24.93	0.81	0.00
ti(Tmax,Rain)	16	14.19	1	0.55
ti(Arable,ALAN)	16	9.22	0.77	0.00
ti(Tmax,ALAN)	16	11.81	0.97	0.02
ti(Woodland,Tmax)	16	8.71	0.94	0.00

Table S6. Deviance Explained (Dev. Exp %) and significance (p values) from Generalized Additive Model fits for the relationships between different covariates and estimated aerial arthropod abundance densities at different heights in the air columns.

Diurnal										
	**100**	**300**	**500**	**700**	**900**	**1100**	**1300**	**1500**	**1700**	**1900**
Dev.Exp.(%)	86.8	84.3	80.2	68.4	49.7	37.1	26.6	29.4	39.4	40.2
s(Tmax)	0.01	0.00	0.00	0.00	0.00	0.03	0.05	0.02	0.04	0.02
s(Rain)	0.80	0.00	0.00	0.00	0.18	0.07	0.12	0.13	0.56	0.19
s(Wind)	0.01	0.00	0.00	0.00	0.00	0.00	0.00	0.00	0.06	0.00
s(Arable)	0.90	0.00	0.00	0.00	0.09	0.00	0.00	0.17	0.40	0.21
s(Urban)	0.64	0.00	0.00	0.07	0.10	0.07	0.76	0.10	0.01	0.48
s(ALAN)	0.00	0.00	0.00	0.00	0.06	0.14	0.34	0.54	0.10	0.30
s(Elevation)	0.10	0.00	0.00	0.00	0.18	0.04	0.68	0.07	0.77	0.06
s(Woodland)	0.00	0.00	0.00	0.00	0.05	0.85	0.30	0.10	0.30	0.09
s(Grassland)	0.56	0.00	0.00	0.00	0.05	0.65	0.40	0.70	0.56	0.99
ti(Tmax,Rain)	0.00	0.01	0.00	0.09	0.89	0.13	0.04	0.05	0.02	0.67
ti(Arable,ALAN)	0.48	0.00	0.00	0.10	0.08	0.10	0.08	0.06	0.10	0.50
ti(Tmax,ALAN)	0.91	0.00	0.00	0.08	0.06	0.23	0.01	0.03	0.14	0.77
ti(Woodland,Tmax)	0.00	0.00	0.00	0.00	0.13	0.02	0.04	0.06	0.16	0.12
**Nocturnal**										
	**100**	**300**	**500**	**700**	**900**	**1100**	**1300**	**1500**	**1700**	**1900**
Dev.Exp.(%)	68.3	59.3	76.4	82.5	88	23.5	37.6	22.3	49.4	56.7
s(Tmax)	0.85	0.00	0.00	0.00	0.01	0.00	0.00	0.00	0.00	0.00
s(Rain)	0.94	0.00	0.00	0.00	0.02	0.00	0.00	0.00	0.00	0.00
s(Wind)	0.92	0.00	0.08	0.02	0.23	0.02	0.00	0.00	0.08	0.01
s(Arable)	0.84	0.00	0.00	0.00	0.03	0.98	0.91	0.70	0.11	0.18
s(Urban)	0.57	0.00	0.01	0.01	0.21	0.06	0.05	0.06	0.01	0.02
s(ALAN)	0.04	0.00	0.00	0.02	0.53	0.53	0.17	0.52	0.09	0.09
s(Elevation)	0.00	0.45	0.00	0.00	0.00	0.08	0.05	0.05	0.00	0.04
s(Woodland)	0.10	0.01	0.00	0.00	0.84	0.09	0.86	0.43	0.13	0.00
s(Grassland)	0.00	0.01	0.00	0.02	0.15	0.12	0.37	0.88	0.05	0.41
ti(Tmax,Rain)	0.18	0.01	0.00	0.00	0.01	0.00	0.00	0.00	0.00	0.00
ti(Arable,ALAN)	0.01	0.00	0.00	0.00	0.48	0.76	0.05	0.09	0.62	0.45
ti(Tmax,ALAN)	0.69	0.01	0.00	0.01	0.00	0.12	0.11	0.20	0.00	0.00
ti(Woodland,Tmax)	0.00	0.03	0.00	0.00	0.00	0.57	0.06	0.05	0.00	0.00

Table S7. Distribution of the extent of land-cover across the Columnar Vertical Profiles (CVPs) used in the study. Values give the proportion of CVPs whose surface area was occupied by each land-cover category at four class thresholds (< 25 %, 26–50 %, 51–75 %, and > 75 %). For example, 62 % of CVPs contained < 25 % woodland, whereas only 4 % were dominated (> 75 %) by woodland.

	Arable	Woodland	Grassland	Urban	Mountains	Coastal	Freshwater	Saltwater
<25% Cover	26	62	24	32	64	100	100	100
26-50% Cover	36	22	32	27	12	0	0	0
51-75% Cover	21	12	25	32	9	0	0	0
>75% Cover	17	4	17	9	15	0	0	0
