# Supplementary material for: Spatio‐Temporal Variation in Aerial Arthropod Abundance Revealed by Weather Radars
Source: Glob Chang Biol. 2025 Oct 27;31(10):e70425. doi: 10.1111/gcb.70425 (PMC12555869; doi:10.1111/gcb.70425)
Supplement: Supplementary file 2 — Data S2: gcb70425‐sup‐0002‐Supinfo2.pdf. [file GCB-31-e70425-s002.pdf]

Dataset S1

Spatio-temporal Variation in Aerial Arthropod Abundance
Revealed by Weather Radars

Authors: Mansi Mungee, Maryna Lukach, Chris Shortall, James R. Bell, Elizabeth J. Duncan, Freya Addison, Lee E. Brown, William E. Kunin, Christopher Hassall, Ryan R. Neely III*

***Corresponding author:** Ryan R. Neely III
Email: R.Neely@leeds.ac.uk

Table: Diurnal: 100m

term	edf	ref.df	statistic	p.value
s(Year)	0.0000682	4	0.0000056	0.3421193
s(Year_factor)	2.9655655	7	1.0477525	0.0140646
s(Radar)	0.0001143	2	0.0000446	0.0000148
s(Year):RadarCastor-Bay	0.0004108	2	0.0001774	0.0562220
s(Year):RadarIngham	0.0000344	2	0.0000012	0.4851861
s(Year):RadarPredannack	0.7211821	2	1.9258570	0.0052920
s(Month)	3.6786823	6	1.7312317	0.0153118
s(x,y)	2.5360372	35	15.4190268	0.0000000
ti(Year,x,y)	15.2008970	92	2.6484530	0.0000000
s(ID)	20.1632000	24	11.7634737	0.0000000
s(Tmax)	1.4594267	29	0.1954215	0.0101840
s(Rain)	0.0000604	29	0.0000003	0.8010955
s(Wind)	0.8345919	29	0.3695637	0.0096502
s(Arable)	0.0000459	27	0.0000001	0.9030559
s(Urban)	0.0000616	29	0.0000004	0.6435942
s(ALAN)	9.7966574	29	11.3368498	0.0031067
s(Elevation)	0.4575935	2	2.0052219	0.0963615
s(Woodland)	1.8285718	29	0.8318769	0.0037425
s(Grassland)	0.0000649	26	0.0000008	0.5634127
ti(Tmax,Rain)	1.4757310	12	1.4414866	0.0002558
ti(Arable,ALAN)	0.0001219	13	0.0000047	0.4781109
ti(Tmax,ALAN)	0.0000607	16	0.0000006	0.9078247
ti(Woodland,Tmax)	4.3450808	16	1.5306242	0.0000107

Table: Diurnal: 300m

term	edf	ref.df	statistic	p.value
s(Year)	0.0000149	4	3.045700e-03	0.0000000
s(Year_factor)	-0.0000653	7	6.434560e-02	0.0000000
s(Radar)	1.8122119	2	4.684697e+03	0.0000000
s(Year):RadarCastor-Bay	0.0001451	2	2.864700e-03	0.0000000
s(Year):RadarIngham	0.0000202	2	2.128000e-04	0.0000000
s(Year):RadarPredannack	0.4920998	2	1.132060e+00	0.0001267
s(Month)	4.5840815	6	3.201966e+03	0.0000000
s(x,y)	0.0008638	37	2.293900e-03	0.0000000
ti(Year,x,y)	17.5573939	88	8.768220e+02	0.0000000
s(ID)	20.0932387	24	1.131885e+02	0.0000000
s(Tmax)	2.1253533	29	6.155229e+02	0.0000000
s(Rain)	-0.0003324	29	5.551800e-02	0.0000000
s(Wind)	0.9962443	29	9.004014e+02	0.0000000
s(Arable)	0.0026167	27	2.987420e-02	0.0000000
s(Urban)	0.5139110	29	7.344966e+01	0.0000000
s(ALAN)	5.3013927	29	1.599316e+02	0.0000000
s(Elevation)	0.0000179	2	1.089000e-04	0.0000583
s(Woodland)	0.0007361	28	3.696480e-02	0.0000000
s(Grassland)	0.0002319	26	1.677740e-02	0.0000000
ti(Tmax,Rain)	63.3108784	8	5.759513e+04	0.0092459
ti(Arable,ALAN)	0.0001551	13	4.364600e-03	0.0000000
ti(Tmax,ALAN)	0.0000163	16	1.714200e-03	0.0000000
ti(Woodland,Tmax)	0.7417714	13	4.089152e+01	0.0000000

Table: Diurnal: 500m

term	edf	ref.df	statistic	p.val
s(Year)	0.0005012	5	0.0000168	0.77736
s(Year_factor)	3.6861041	8	47.1813575	0.00000
s(Radar)	11.3316459	14	23.5752857	0.00000
s(Year):RadarCastor-Bay	0.0085485	5	0.0013361	0.34736
s(Year):RadarChenies	3.2836801	5	49.2838444	0.00000
s(Year):RadarCobbacombe-Cross	2.2966737	4	3.9356604	0.00011
s(Year):RadarCrug-y-Gorllwyn	0.0015466	4	0.0000788	0.73196
s(Year):RadarDean-Hill	0.0029852	5	0.0001490	0.64622
s(Year):RadarDruim-a-Starraig	0.0037185	4	0.0003120	0.54244
s(Year):RadarHameldon-Hill	3.6487318	5	23.9620636	0.00000
s(Year):RadarHigh-Moorsley	3.1681673	5	26.4052844	0.00000
s(Year):RadarHill-of-Dudwick	3.9146256	5	46.9606405	0.00000
s(Year):RadarHolehead	0.0127643	4	0.0026335	0.26256
s(Year):RadarIngham	0.0101410	4	0.0017515	0.41401
s(Year):RadarMunduff-Hill	2.9135350	4	16.2324902	0.00000
s(Year):RadarPredannack	1.6436008	5	0.8546077	0.04460
s(Year):RadarThurnham	3.0876934	5	22.2422711	0.00000
s(Month)	5.6821773	7	63.4611690	0.00000
s(x,y)	82.8234681	99	7714.5680335	0.00008
ti(Year,x,y)	73.0492599	96	19.4206619	0.00000
s(ID)	93.9616082	131	4.2600236	0.00000
s(Tmax)	20.8189326	29	27.5233280	0.00000
s(Rain)	16.1452984	29	11.8042903	0.00000
s(Wind)	14.4897207	29	4.4896826	0.00000
s(Arable)	24.4262546	29	15.7533607	0.00000
s(Urban)	19.6191744	29	10.8718544	0.00000
s(ALAN)	24.9549644	29	12.0030779	0.00000
s(Elevation)	1.8375617	2	331.3681029	0.00000
s(Woodland)	12.9201969	29	9.3469208	0.00000
s(Grassland)	21.6999283	29	19.8882306	0.00000
ti(Tmax,Rain)	12.5803445	16	36.9306338	0.00000
ti(Arable,ALAN)	10.9867288	16	4.4557407	0.00000
ti(Tmax,ALAN)	10.2587161	16	10.3037219	0.00000
ti(Woodland,Tmax)	9.5196989	16	5.0900613	0.00000

Table: Diurnal: 700m

term	edf	ref.df	statistic	p.va[illegible]
s(Year)	0.0000130	5	0.0000075	0.0052[illegible]
s(Year_factor)	3.4461412	8	8.2218461	0.0000[illegible]
s(Radar)	5.3595650	10	7.8891504	-0.0000[illegible]
s(Year):RadarCastor-Bay	0.9357010	5	3.1526910	0.0000[illegible]
s(Year):RadarChenies	3.5694931	5	118.9120779	0.0000[illegible]
s(Year):RadarCobbacombe-Cross	0.0015273	4	0.0003833	0.2013[illegible]
s(Year):RadarDean-Hill	0.0062530	5	0.0009942	0.2384[illegible]
s(Year):RadarDruim-a-Starraig	0.0007442	3	0.0000226	0.7062[illegible]
s(Year):RadarHigh-Moorsley	3.6797452	5	14.5122681	0.0000[illegible]
s(Year):RadarHill-of-Dudwick	1.8653321	3	15.5931154	0.0000[illegible]
s(Year):RadarIngham	0.9447097	4	4.7563924	0.0000[illegible]
s(Year):RadarPredannack	2.2736801	5	1.9725974	0.0063[illegible]
s(Year):RadarThurnham	3.4822125	5	9.1292885	0.0000[illegible]
s(Month)	5.7098287	7	33.1068654	0.0000[illegible]
s(x,y)	62.6474290	98	1892.8488200	0.1585[illegible]
ti(Year,x,y)	60.7651577	96	13.9765022	0.0008[illegible]
s(ID)	81.0041280	114	9.7876935	0.0000[illegible]
s(Tmax)	16.9785984	29	10.6452158	0.0000[illegible]
s(Rain)	12.8422581	29	4.9624279	0.0000[illegible]
s(Wind)	23.0855397	29	9.8271773	0.0000[illegible]
s(Arable)	25.8277846	29	25.9608460	0.0034[illegible]
s(Urban)	22.2310610	29	22.6866950	0.0664[illegible]
s(ALAN)	24.9735372	29	13.4227289	0.0013[illegible]
s(Elevation)	1.9102932	2	95.3400849	0.0000[illegible]
s(Woodland)	24.7303100	29	19.1890198	0.0005[illegible]
s(Grassland)	18.0571460	29	7.0260014	0.0000[illegible]
ti(Tmax,Rain)	12.1145888	16	18.4112585	0.0876[illegible]
ti(Arable,ALAN)	15.7556162	16	19.9872821	0.0954[illegible]
ti(Tmax,ALAN)	11.5250227	16	5.8938329	0.0836[illegible]
ti(Woodland,Tmax)	9.3001541	16	6.4765914	0.0045[illegible]

Table: Diurnal: 900m

term	edf	ref.df	statistic	p.val[illegible]
s(Year)	0.0000064	5	0.0000011	0.24553[illegible]
s(Year_factor)	5.6225403	8	76.0170943	0.00000[illegible]
s(Radar)	9.0253579	15	5.6157486	0.00000[illegible]
s(Year):RadarCastor-Bay	0.0014046	5	0.0001083	0.57498[illegible]
s(Year):RadarChenies	3.5944993	5	35.0347534	0.00231[illegible]
s(Year):RadarClee-Hill	0.6709031	1	10.0475096	0.05671[illegible]
s(Year):RadarCobbacombe-Cross	2.6822247	4	7.5278946	0.00000[illegible]
s(Year):RadarCrug-y-Gorllwyn	2.4879667	4	4.9501602	0.00001[illegible]
s(Year):RadarDean-Hill	0.0024322	5	0.0001680	0.61181[illegible]
s(Year):RadarDruim-a-Starraig	0.0043491	3	0.0004988	0.48128[illegible]
s(Year):RadarHameldon-Hill	3.8999027	5	48.3983205	0.00000[illegible]
s(Year):RadarHigh-Moorsley	1.3474808	5	0.9347454	0.02741[illegible]
s(Year):RadarHill-of-Dudwick	3.2790160	4	72.6850641	0.00000[illegible]
s(Year):RadarHolehead	2.4971802	4	17.4246176	0.00000[illegible]
s(Year):RadarIngham	2.3508604	4	6.3617334	0.00000[illegible]
s(Year):RadarMunduff-Hill	3.0993203	4	113.0096170	0.00000[illegible]
s(Year):RadarPredannack	0.0000324	5	0.0000149	0.07527[illegible]
s(Year):RadarThurnham	3.6970829	5	10.1501544	0.00000[illegible]
s(Month)	5.8558420	7	175.7135409	0.00000[illegible]
s(x,y)	63.9156866	99	246.1564544	0.16857[illegible]
ti(Year,x,y)	68.2218630	96	126.6381800	0.00000[illegible]
s(ID)	95.8885039	131	4.4118347	0.00000[illegible]
s(Tmax)	19.4425969	29	27.9914215	0.00183[illegible]
s(Rain)	15.3463059	29	14.9160605	0.18274[illegible]
s(Wind)	14.8482666	29	5.6957098	-0.00000[illegible]
s(Arable)	12.4306700	29	3.2845760	0.08727[illegible]
s(Urban)	10.2627832	29	4.0990549	0.09826[illegible]
s(ALAN)	14.7396911	29	4.3045889	0.05671[illegible]
s(Elevation)	0.9795193	2	63.7984833	0.17789[illegible]
s(Woodland)	0.8172344	29	0.3337657	0.05124[illegible]
s(Grassland)	16.0602780	29	7.6094081	0.05283[illegible]
ti(Tmax,Rain)	13.4076167	16	37.8985655	0.88760[illegible]
ti(Arable,ALAN)	10.9434883	16	5.3022192	0.08127[illegible]
ti(Tmax,ALAN)	9.2926169	16	5.3258171	0.06038[illegible]
ti(Woodland,Tmax)	11.5641236	16	6.8814758	0.12987[illegible]

Table: Diurnal: 1100m

term	edf	ref.df	statistic	p.valu
s(Year)	0.0005686	5	0.0000348	0.540005
s(Year_factor)	4.6974527	8	74.7716970	0.000000
s(Radar)	3.5612658	15	0.5175697	0.000000
s(Year):RadarCastor-Bay	0.0026446	5	0.0004064	0.108253
s(Year):RadarChenies	3.4194156	5	22.1451297	0.000449
s(Year):RadarClee-Hill	0.0016218	1	0.0003662	0.536417
s(Year):RadarCobbacombe-Cross	3.0275387	4	7.1017299	0.000000
s(Year):RadarCrug-y-Gorllwyn	2.4577337	4	2.5288082	0.000905
s(Year):RadarDean-Hill	0.0129023	5	0.0025083	0.189678
s(Year):RadarDruim-a-Starraig	0.0014432	4	0.0000418	0.609307
s(Year):RadarHameldon-Hill	3.8813862	5	42.0595097	0.000000
s(Year):RadarHigh-Moorsley	3.2812386	5	4.3353608	0.000016
s(Year):RadarHill-of-Dudwick	2.9392033	3	46.9976142	0.000000
s(Year):RadarHolehead	1.8860291	4	1.5914045	0.014398
s(Year):RadarIngham	1.5268578	4	1.4145939	0.001842
s(Year):RadarMunduff-Hill	3.1157012	4	8.7936087	0.000000
s(Year):RadarPredannack	0.0020701	5	0.0000670	0.717302
s(Year):RadarThurnham	3.6794529	5	6.6591720	0.000036
s(Month)	5.9215252	7	403.2764858	0.000000
s(x,y)	31.6958258	99	2.6780803	0.425439
ti(Year,x,y)	50.7211397	96	10.1679079	0.000006
s(ID)	56.0376521	121	1.1895556	0.000000
s(Tmax)	20.6130848	29	26.9810001	0.030091
s(Rain)	10.0735398	29	11.0454413	0.066719
s(Wind)	12.0763387	29	3.9515275	0.000015
s(Arable)	8.5618801	29	0.8779820	0.002344
s(Urban)	26.3793343	29	7.4633079	0.072990
s(ALAN)	20.0459169	29	4.5374427	0.144200
s(Elevation)	0.7347538	2	2.1152576	0.044093
s(Woodland)	0.0018264	29	0.0000111	0.851505
s(Grassland)	12.0522807	29	3.1077618	0.652889
ti(Tmax,Rain)	13.6365089	16	47.5571561	0.129873
ti(Arable,ALAN)	11.6717821	16	4.6750033	0.098111
ti(Tmax,ALAN)	8.6145454	16	4.0448585	0.226881
ti(Woodland,Tmax)	4.4424709	16	0.6431370	0.023004

Table: Diurnal: 1300m

term	edf	ref.df	statistic	p.valu[illegible]
s(Year)	0.0011649	5	0.0000534	0.689849[illegible]
s(Year_factor)	4.2385238	8	70.8507902	0.000000[illegible]
s(Radar)	11.6597446	15	5.7375847	0.000000[illegible]
s(Year):RadarCastor-Bay	2.7212278	5	2.4768849	0.000256[illegible]
s(Year):RadarChenies	3.3393351	5	35.6042485	0.000016[illegible]
s(Year):RadarClee-Hill	0.0004550	1	0.0001193	0.603236[illegible]
s(Year):RadarCobbacombe-Cross	2.7410182	4	8.7224259	0.000000[illegible]
s(Year):RadarCrug-y-Gorllwyn	2.6254554	4	5.3113823	0.000018[illegible]
s(Year):RadarDean-Hill	0.0024002	5	0.0003643	0.284744[illegible]
s(Year):RadarDruim-a-Starraig	0.0002982	4	0.0001449	0.051032[illegible]
s(Year):RadarHameldon-Hill	3.8634426	5	30.6485615	0.000000[illegible]
s(Year):RadarHigh-Moorsley	3.4166155	5	3.7341172	0.000334[illegible]
s(Year):RadarHill-of-Dudwick	3.8882347	5	41.4555727	0.000000[illegible]
s(Year):RadarHolehead	0.8291981	4	2.1393090	0.003061[illegible]
s(Year):RadarIngham	2.4408313	4	3.3136414	0.000643[illegible]
s(Year):RadarMunduff-Hill	2.9668092	4	13.5500378	0.000229[illegible]
s(Year):RadarPredannack	0.0003952	5	0.0001099	0.120531[illegible]
s(Year):RadarThurnham	3.9267767	5	39.1343059	0.000000[illegible]
s(Month)	5.9072933	7	277.5490517	0.000000[illegible]
s(x,y)	18.0378705	99	3.8310685	0.341307[illegible]
ti(Year,x,y)	55.1460091	96	10.1324139	0.000017[illegible]
s(ID)	41.2605943	109	0.8030676	0.000000[illegible]
s(Tmax)	19.2192652	29	46.4689927	0.048890[illegible]
s(Rain)	8.4977916	29	3.6685279	0.120091[illegible]
s(Wind)	14.2495265	29	3.5943795	0.000076[illegible]
s(Arable)	4.8494654	29	0.5801231	0.001653[illegible]
s(Urban)	25.5611945	29	8.3439705	0.763390[illegible]
s(ALAN)	7.8686208	29	1.2617804	0.341003[illegible]
s(Elevation)	0.0022599	2	0.0003440	0.684790[illegible]
s(Woodland)	0.1286476	29	0.0047607	0.304553[illegible]
s(Grassland)	0.0055367	29	0.0001488	0.398163[illegible]
ti(Tmax,Rain)	11.6864122	16	49.9555977	0.040091[illegible]
ti(Arable,ALAN)	1.5392136	16	0.2145875	0.079773[illegible]
ti(Tmax,ALAN)	10.0911506	16	2.4753533	0.012008[illegible]
ti(Woodland,Tmax)	9.2622266	16	6.0742183	0.040019[illegible]

Table: Diurnal: 1500m

term	edf	ref.df	statistic	p.valu[illegible]
s(Year)	0.0020499	5	0.0002546	0.314246[illegible]
s(Year_factor)	4.0469730	8	53.7304684	0.000000[illegible]
s(Radar)	8.1855958	15	1.9869351	0.000000[illegible]
s(Year):RadarCastor-Bay	2.3186863	5	1.4015986	0.003892[illegible]
s(Year):RadarChenies	0.0034434	5	0.0004191	0.380750[illegible]
s(Year):RadarClee-Hill	0.0004031	1	0.0001154	0.563715[illegible]
s(Year):RadarCobbacombe-Cross	2.9108469	4	10.8273665	0.000000[illegible]
s(Year):RadarCrug-y-Gorllwyn	3.0597493	4	7.7737105	0.000002[illegible]
s(Year):RadarDean-Hill	0.0022341	5	0.0003466	0.342514[illegible]
s(Year):RadarDruim-a-Starraig	0.0000886	4	0.0000011	0.898989[illegible]
s(Year):RadarHameldon-Hill	3.8795886	5	31.9663951	0.000000[illegible]
s(Year):RadarHigh-Moorsley	3.5632697	5	4.3911211	0.000044[illegible]
s(Year):RadarHill-of-Dudwick	3.6960136	5	22.7539950	0.000000[illegible]
s(Year):RadarHolehead	0.9245806	4	5.1784954	0.000019[illegible]
s(Year):RadarIngham	3.0806747	4	8.1865471	0.000002[illegible]
s(Year):RadarMunduff-Hill	2.8703750	4	10.5382495	0.000096[illegible]
s(Year):RadarPredannack	0.0004421	5	0.0001260	0.122023[illegible]
s(Year):RadarThurnham	3.8518914	5	38.1814707	0.000000[illegible]
s(Month)	5.9507858	7	222.4430055	0.000000[illegible]
s(x,y)	30.4702713	99	1.2032194	0.888034[illegible]
ti(Year,x,y)	59.7527596	96	11.1318016	0.006578[illegible]
s(ID)	13.8897004	115	0.1488029	0.059210[illegible]
s(Tmax)	20.6100063	29	55.8830964	0.020098[illegible]
s(Rain)	9.9850780	29	16.4062483	0.130921[illegible]
s(Wind)	1.9820883	29	1.9798440	0.000277[illegible]
s(Arable)	0.8755106	29	0.0408243	0.172275[illegible]
s(Urban)	25.7464533	29	7.0257908	0.102889[illegible]
s(ALAN)	3.3297652	29	0.5445063	0.542228[illegible]
s(Elevation)	0.7741525	2	1.8374123	0.072671[illegible]
s(Woodland)	3.2313107	29	0.7139663	0.100021[illegible]
s(Grassland)	6.5259288	29	0.6821720	0.700709[illegible]
ti(Tmax,Rain)	14.4689952	16	59.4578168	0.053890[illegible]
ti(Arable,ALAN)	4.0586671	16	0.5852268	0.061093[illegible]
ti(Tmax,ALAN)	9.5649773	16	3.3331410	0.032098[illegible]
ti(Woodland,Tmax)	11.4101567	16	7.9601396	0.056782[illegible]

Table: Diurnal: 1700m

term	edf	ref.df	statistic	p.value
s(Year)	0.0005689	5	0.0000510	0.3871159
s(Year_factor)	4.2538131	7	33.8007470	0.0000000
s(Radar)	0.0005945	15	0.0000218	0.0843420
s(Year):RadarCastor-Bay	2.0967747	5	1.1601494	0.0042731
s(Year):RadarChenies	0.0004986	5	0.0000388	0.4496546
s(Year):RadarClee-Hill	0.8431005	1	5.3965852	0.0012950
s(Year):RadarCobbacombe-Cross	3.5491906	4	14.0739944	0.0000000
s(Year):RadarCrug-y-Gorllwyn	1.8583432	3	1.9178973	0.0059942
s(Year):RadarDean-Hill	1.4665379	5	0.7959029	0.0302829
s(Year):RadarDruim-a-Starraig	0.0017540	4	0.0001941	0.3277234
s(Year):RadarHameldon-Hill	3.7662336	5	24.1659786	0.0000000
s(Year):RadarHigh-Moorsley	0.5098333	5	0.1592076	0.0950858
s(Year):RadarHill-of-Dudwick	3.2131394	5	4.7844510	0.0000118
s(Year):RadarHolehead	2.1803428	4	5.6347177	0.0000187
s(Year):RadarIngham	0.0001485	4	0.0000139	0.4624290
s(Year):RadarMunduff-Hill	3.2998199	4	11.7247578	0.0000000
s(Year):RadarPredannack	1.7456935	5	1.0936213	0.0058430
s(Year):RadarThurnham	3.1309963	5	7.6129789	0.0000000
s(Month)	5.9565490	7	94.4205811	0.0000000
s(x,y)	28.4888716	99	1.8187646	0.0000000
ti(Year,x,y)	62.6324463	96	10.7616126	0.0015578
s(ID)	0.0026010	121	0.0000182	0.7992735
s(Tmax)	20.9131174	29	72.8903345	0.0432789
s(Rain)	21.6157814	29	7.1433896	0.5636780
s(Wind)	4.5964858	29	2.5421951	0.0582400
s(Arable)	3.4395845	29	0.7968269	0.3977689
s(Urban)	0.8774527	29	0.2519885	0.0108826
s(ALAN)	0.8995034	29	0.3475224	0.0960004
s(Elevation)	1.6167103	2	7.4162213	0.7652608
s(Woodland)	0.0081199	29	0.0002636	0.2997184
s(Grassland)	0.0019298	29	0.0000325	0.5607223
ti(Tmax,Rain)	13.0662456	16	39.5659289	0.0233000
ti(Arable,ALAN)	0.9216092	16	0.7147299	0.1001910
ti(Tmax,ALAN)	7.0947544	16	2.4714910	0.1387914
ti(Woodland,Tmax)	7.7576991	16	2.7449223	0.1556789

Table: Diurnal: 1900m

term	edf	ref.df	statistic	p.valu
s(Year)	0.9584445	5	0.5314232	0.027998
s(Year_factor)	3.8633515	7	18.5894541	0.000000
s(Radar)	0.0014895	15	0.0000582	0.063094
s(Year):RadarCastor-Bay	0.0002899	5	0.0000900	0.015333
s(Year):RadarChenies	0.0015481	5	0.0001519	0.435322
s(Year):RadarClee-Hill	0.0006404	1	0.0010010	0.081660
s(Year):RadarCobbacombe-Cross	3.1232693	4	7.7984378	0.000000
s(Year):RadarCrug-y-Gorllwyn	2.1918000	3	3.5847260	0.000252
s(Year):RadarDean-Hill	0.0099182	5	0.0019425	0.268108
s(Year):RadarDruim-a-Starraig	0.0000879	4	0.0000614	0.001690
s(Year):RadarHameldon-Hill	3.6635881	5	11.1894044	0.000000
s(Year):RadarHigh-Moorsley	1.9596457	5	1.3823216	0.002056
s(Year):RadarHill-of-Dudwick	3.2553514	5	6.9018862	0.000000
s(Year):RadarHolehead	1.8277280	4	1.7573235	0.000434
s(Year):RadarIngham	0.0007301	4	0.0000497	0.582196
s(Year):RadarMunduff-Hill	3.4457219	4	8.9142078	0.000000
s(Year):RadarPredannack	0.0003284	5	0.0000567	0.270340
s(Year):RadarThurnham	3.1815873	5	5.5488758	0.000001
s(Month)	5.9651449	7	107.7443200	0.000000
s(x,y)	32.0569154	99	1.5935448	0.000000
ti(Year,x,y)	62.2890119	96	8.6910268	0.000009
s(ID)	8.7036794	124	0.0776225	0.197750
s(Tmax)	21.9048509	29	92.9539342	0.019987
s(Rain)	15.6457986	29	8.9561812	0.189554
s(Wind)	10.8752167	29	2.6954694	0.001049
s(Arable)	3.9162985	29	0.3842198	0.205986
s(Urban)	21.3270720	29	3.6281629	0.477812
s(ALAN)	2.3428259	29	0.4505168	0.300639
s(Elevation)	0.8516964	2	3.0261122	0.062340
s(Woodland)	0.7641939	29	0.1149474	0.092360
s(Grassland)	13.3359849	29	1.4182800	0.987009
ti(Tmax,Rain)	12.5393150	16	41.8590124	0.665470
ti(Arable,ALAN)	2.2324118	16	0.6207265	0.502651
ti(Tmax,ALAN)	6.6868711	16	1.6288377	0.766520
ti(Woodland,Tmax)	9.4363264	16	3.5863452	0.122901

Table: Nocturnal: 100m

term	edf	ref.df	statistic	p.value
s(Year)	0.0000630	4	0.0000009	0.9227720
s(Year_factor)	4.7376009	7	3.7600827	0.0000314
s(Radar)	0.0000342	2	0.0000070	0.0024506
s(Year):RadarCastor-Bay	0.5563568	2	1.3073197	0.1004427
s(Year):RadarIngham	0.0000622	2	0.0000022	0.7841925
s(Year):RadarPredannack	0.9142584	2	37.0707969	0.0004543
s(Month_abb)	3.2811054	6	3.6249504	0.0000567
s(x,y)	3.0017264	34	13.6378575	0.0000000
ti(Year,x,y)	0.7452816	73	0.2541090	0.0540204
s(ID)	17.2939685	23	4.7481972	0.0000000
s(Tmax)	0.0000526	9	0.0000008	0.8481613
s(Rain)	0.0000513	9	0.0000003	0.9410238
s(Wind)	0.0000424	9	0.0000004	0.9205089
s(Arable)	0.0000563	9	0.0000005	0.8373292
s(Urban)	0.0000876	9	0.0000024	0.5701052
s(ALAN)	1.1553903	9	0.5618914	0.0378229
s(Elevation)	0.8566476	2	13.2988955	0.0024786
s(Woodland)	0.6225055	9	0.1824275	0.0974208
s(Grassland)	1.9898905	9	1.4748304	0.0006786
ti(Tmax,Rain)	0.4275060	16	0.0740878	0.1790209
ti(Arable,ALAN)	1.0438006	13	0.5643653	0.0094647
ti(Tmax,ALAN)	0.0001138	16	0.0000031	0.6881827
ti(Woodland,Tmax)	1.1649066	16	1.7311398	0.0019811

Table: Nocturnal: 300m

term	edf	ref.df	statistic	p.value
s(Year)	0.0000590	4	0.0000108	0.1821543
s(Year_factor)	2.5814139	7	0.9966099	0.0130170
s(Radar)	0.0000411	2	0.0000013	0.8407717
s(Year):RadarCastor-Bay	0.0001015	2	0.0000357	0.1852418
s(Year):RadarIngham	0.0001257	2	0.0000575	0.1026523
s(Year):RadarPredannack	0.0000671	2	0.0000158	0.2785375
s(Month_abb)	4.6302790	6	7.8480056	0.0000000
s(x,y)	0.0000813	36	0.0000001	0.9014620
ti(Year,x,y)	16.5926955	87	1.6695720	0.0000101
s(ID)	9.5262005	23	0.9323395	0.0010159
s(Tmax)	0.0193629	29	0.0002587	0.0000999
s(Rain)	1.8280360	29	0.4379676	0.0037985
s(Wind)	0.0000417	29	0.0000002	0.0000623
s(Arable)	0.0000352	26	0.0000004	0.0005669
s(Urban)	0.0000674	28	0.0000029	0.0002535
s(ALAN)	4.2095302	9	5.2147696	0.0000008
s(Elevation)	0.0000439	2	0.0000125	0.4532163
s(Woodland)	0.0000149	21	0.0000029	0.0123141
s(Grassland)	0.0000597	9	0.0000059	0.0060398
ti(Tmax,Rain)	0.0205031	16	0.0004039	0.0077163
ti(Arable,ALAN)	1.4051946	14	0.1650525	0.0005467
ti(Tmax,ALAN)	0.7632271	16	0.2371422	0.0087216
ti(Woodland,Tmax)	0.8211364	14	0.3066411	0.0344760

Table: Nocturnal: 500m

term	edf	ref.df	statistic	p.value
s(Year)	0.0016505	5	0.0001793	0.0000128
s(Year_factor)	3.2726898	7	13.1321131	0.0000000
s(Radar)	10.7455284	13	11.7891466	0.0000000
s(Year):RadarCastor-Bay	1.4341907	5	0.5878857	0.0337299
s(Year):RadarChenies	0.0021332	5	0.0003030	0.3881276
s(Year):RadarCobbacombe-Cross	1.9882278	4	2.1079262	0.0033191
s(Year):RadarCrug-y-Gorllwyn	1.9269010	4	3.5838277	0.0000479
s(Year):RadarDean-Hill	0.0014699	5	0.0000752	0.6619544
s(Year):RadarDruim-a-Starraig	2.4063315	4	5.6977755	0.0000000
s(Year):RadarHameldon-Hill	0.0024797	5	0.0003075	0.3076365
s(Year):RadarHigh-Moorsley	1.8903882	5	1.1430154	0.0048626
s(Year):RadarHill-of-Dudwick	3.9252216	5	30.6868626	0.0000000
s(Year):RadarHolehead	3.0685623	4	9.9147688	0.0000000
s(Year):RadarIngham	1.2812935	4	1.6184444	0.0035564
s(Year):RadarMunduff-Hill	1.5545614	4	1.1977843	0.0243419
s(Year):RadarPredannack	2.7555292	5	5.0542836	0.0000083
s(Year):RadarThurnham	3.2500322	5	58.6225385	0.0000000
s(Month_abb)	5.6953177	6	63.6556827	0.0000000
s(x,y)	71.2697500	99	2649.3287170	0.0004499
ti(Year,x,y)	63.5513136	96	12.7401397	0.0000003
s(ID)	84.9053631	129	2.8629631	0.0000000
s(Tmax)	19.7715795	29	14.4131543	0.0000000
s(Rain)	11.2699975	29	12.3963196	0.0000000
s(Wind)	3.7689617	29	0.5573156	0.0786830
s(Arable)	16.1434417	29	3.7809718	0.0000000
s(Urban)	0.6000691	29	0.0880514	0.0071735
s(ALAN)	24.8251685	29	9.9930230	0.0000000
s(Elevation)	1.7748545	2	189.2172170	0.0000000
s(Woodland)	16.2339518	29	4.5760134	0.0000000
s(Grassland)	24.9329660	29	11.9231839	0.0000000
ti(Tmax,Rain)	14.1851687	16	22.6028830	0.0000000
ti(Arable,ALAN)	9.2158532	16	4.1947975	0.0000000
ti(Tmax,ALAN)	11.8092991	16	4.2735208	0.0000000
ti(Woodland,Tmax)	8.7143637	16	3.9068988	0.0000000

Table: Nocturnal: 700m

term	edf	ref.df	statistic	p.value
s(Year)	2.6774545	5	4.6193939	0.0009495
s(Year_factor)	1.7632199	7	1.9232570	0.0000774
s(Radar)	0.0002964	9	0.0000046	0.7573859
s(Year):RadarCastor-Bay	0.0000805	5	0.0000060	0.4916649
s(Year):RadarChenies	2.2182153	5	1.2778318	0.0079447
s(Year):RadarCobbacombe-Cross	0.0009695	2	0.0007119	0.1441499
s(Year):RadarDean-Hill	0.0059452	5	0.0008280	0.2872146
s(Year):RadarDruim-a-Starraig	1.3675500	3	1.6331500	0.0016812
s(Year):RadarHigh-Moorsley	3.3490408	5	5.1897602	0.0000000
s(Year):RadarHill-of-Dudwick	0.0002160	2	0.0000048	0.6722513
s(Year):RadarIngham	0.0012376	4	0.0001892	0.3920229
s(Year):RadarPredannack	1.3653005	5	0.8824364	0.0075490
s(Year):RadarThurnham	1.1449258	5	5.8300647	0.0000000
s(Month_abb)	5.3695573	6	29.5024370	0.0000000
s(x,y)	50.7403642	99	15.9598294	0.0000000
ti(Year,x,y)	55.6823679	96	6.5535613	0.0000000
s(ID)	70.8156347	112	4.4742986	0.0000000
s(Tmax)	10.3802953	29	4.8063299	0.0012459
s(Rain)	2.2600764	29	0.8233427	0.0000014
s(Wind)	1.4018039	29	0.3914027	0.0228145
s(Arable)	20.2914190	29	6.8843141	0.0000000
s(Urban)	12.9946514	29	4.7930472	0.0134560
s(ALAN)	8.5249911	29	2.0992194	0.0231190
s(Elevation)	1.8807259	2	49.1613845	0.0000048
s(Woodland)	18.2670959	29	3.8483215	0.0000005
s(Grassland)	12.1654993	29	3.2179833	0.0231121
ti(Tmax,Rain)	9.1979662	16	2.9796347	0.0000000
ti(Arable,ALAN)	10.6995602	16	4.5234902	0.0000000
ti(Tmax,ALAN)	5.9463832	16	0.9420819	0.0071724
ti(Woodland,Tmax)	9.6244169	16	4.5709598	0.0000000

Table: Nocturnal: 900m

term	edf	ref.df	statistic	p.value
s(Year)	0.0018217	5	0.0002059	0.2274619
s(Year_factor)	3.7864572	7	32.0098198	0.0000000
s(Radar)	4.3070230	13	1.3094386	0.0000139
s(Year):RadarCastor-Bay	0.6974972	5	0.4705101	0.0000133
s(Year):RadarChenies	0.0007712	5	0.0000392	0.7003587
s(Year):RadarCobbacombe-Cross	0.7873849	4	0.9593690	0.0135946
s(Year):RadarCrug-y-Gorllwyn	2.0159475	4	2.0516654	0.0016652
s(Year):RadarDean-Hill	2.6686827	5	2.3792442	0.0005533
s(Year):RadarDruim-a-Starraig	0.7867150	3	1.4539041	0.0020100
s(Year):RadarHameldon-Hill	0.9429054	5	3.2534551	0.0000000
s(Year):RadarHigh-Moorsley	0.0019724	5	0.0002152	0.3119618
s(Year):RadarHill-of-Dudwick	3.6925939	4	51.0236681	0.0000000
s(Year):RadarHolehead	2.9040146	4	9.2538222	0.0000000
s(Year):RadarIngham	0.9233532	4	3.1821322	0.0000007
s(Year):RadarMunduff-Hill	3.2467067	4	10.3501853	0.0000014
s(Year):RadarPredannack	3.7223904	5	5.8000461	0.0000065
s(Year):RadarThurnham	3.7446683	5	13.6807459	0.0000000
s(Month_abb)	5.6883393	6	68.6001939	0.0000000
s(x,y)	50.7522538	99	15.9026075	0.6796428
ti(Year,x,y)	46.6313731	96	5.8319189	0.0013982
s(ID)	93.7508756	126	3.3700025	0.0000000
s(Tmax)	18.2271410	29	17.4282809	0.0111770
s(Rain)	15.3971898	29	16.0143268	0.0221000
s(Wind)	0.2686680	29	0.0153900	0.2250756
s(Arable)	2.5524966	29	0.4221356	0.0298193
s(Urban)	0.2755486	29	0.0266662	0.2134352
s(ALAN)	0.0015906	29	0.0000314	0.5302169
s(Elevation)	0.9826916	2	75.9427118	0.0000000
s(Woodland)	0.0011349	29	0.0000051	0.8411230
s(Grassland)	0.7064312	29	0.0476404	0.1537321
ti(Tmax,Rain)	14.1306720	16	26.6743200	0.0115580
ti(Arable,ALAN)	0.0043504	16	0.0001819	0.4786023
ti(Tmax,ALAN)	7.8864068	16	2.4945352	0.0000018
ti(Woodland,Tmax)	5.3771896	16	3.6307700	0.0000000

Table: Nocturnal: 1100m

term	edf	ref.df	statistic	p.value
s(Year)	0.0016957	5	0.0002809	0.1181067
s(Year_factor)	3.6997249	7	14.3332605	0.0000000
s(Radar)	0.7263427	13	0.0719325	0.0117307
s(Year):RadarCastor-Bay	0.0051922	5	0.0010582	0.0428105
s(Year):RadarChenies	0.0004050	5	0.0000163	0.7651393
s(Year):RadarCobbacombe-Cross	0.0008183	4	0.0000304	0.7126573
s(Year):RadarCrug-y-Gorllwyn	1.5493977	4	1.1684214	0.0001591
s(Year):RadarDean-Hill	1.1113031	5	0.5374492	0.0113596
s(Year):RadarDruim-a-Starraig	1.9893631	4	1.8122332	0.0000075
s(Year):RadarHameldon-Hill	0.8438829	5	1.1111397	0.0000028
s(Year):RadarHigh-Moorsley	0.8961466	5	0.3005970	0.0129039
s(Year):RadarHill-of-Dudwick	1.9726340	2	73.3430762	0.0000000
s(Year):RadarHolehead	0.0015834	4	0.0002020	0.4145666
s(Year):RadarIngham	2.7112750	4	8.5544376	0.0000000
s(Year):RadarMunduff-Hill	0.0013785	4	0.0001559	0.5163595
s(Year):RadarPredannack	2.5069816	5	2.8721303	0.0000097
s(Year):RadarThurnham	4.8482577	5	18.4752501	0.0000000
s(Month_abb)	5.4276746	6	37.8297242	0.0000000
s(x,y)	3.4640692	99	0.1293451	0.0147650
ti(Year,x,y)	44.4696581	96	6.5229882	0.0000075
s(ID)	35.9288593	109	0.6146330	0.0000081
s(Tmax)	19.4093939	29	13.0394242	0.0000000
s(Rain)	11.7056787	29	13.5829450	0.0000000
s(Wind)	1.6868582	29	0.3965151	0.0223462
s(Arable)	0.0004537	29	0.0000009	0.9783909
s(Urban)	0.9314797	29	0.6343556	0.0613000
s(ALAN)	0.0010703	29	0.0000217	0.5270031
s(Elevation)	1.2701099	2	3.8262438	0.0801551
s(Woodland)	2.4786016	29	0.6333069	0.0930004
s(Grassland)	0.5497035	29	0.0490771	0.1157999
ti(Tmax,Rain)	14.3564640	16	24.7306395	0.0000000
ti(Arable,ALAN)	0.0019290	16	0.0000556	0.7550669
ti(Tmax,ALAN)	7.1261589	16	3.5539567	0.1190221
ti(Woodland,Tmax)	12.2844094	16	8.9643642	0.5733901

Table: Nocturnal: 1300m

term	edf	ref.df	statistic	p.value
s(Year)	0.9133342	5	2.4750280	0.0000000
s(Year_factor)	3.7338478	7	9.4847348	0.0000000
s(Radar)	0.0016398	13	0.0000791	0.1458398
s(Year):RadarCastor-Bay	0.0005220	5	0.0000513	0.1397047
s(Year):RadarChenies	0.0022017	5	0.0003213	0.2886948
s(Year):RadarCobbacombe-Cross	0.0004403	4	0.0000283	0.4945066
s(Year):RadarCrug-y-Gorllwyn	2.0607594	4	1.7813320	0.0000708
s(Year):RadarDean-Hill	0.0013824	5	0.0001446	0.3536060
s(Year):RadarDruim-a-Starraig	1.3894432	4	0.8745756	0.0013569
s(Year):RadarHameldon-Hill	0.6471498	5	0.1746044	0.0036422
s(Year):RadarHigh-Moorsley	0.8376493	5	0.2388786	0.0188219
s(Year):RadarHill-of-Dudwick	4.9323252	5	79.7283297	0.0000000
s(Year):RadarHolehead	1.8219362	4	1.6124124	0.0027817
s(Year):RadarIngham	1.6251423	3	1.5480979	0.0013730
s(Year):RadarMunduff-Hill	0.0006423	4	0.0000333	0.6646912
s(Year):RadarPredannack	0.9452923	5	3.4608053	0.0000000
s(Year):RadarThurnham	4.8715749	5	16.2986217	0.0000000
s(Month_abb)	5.5107386	6	25.0131607	0.0000000
s(x,y)	8.5503070	99	0.2821663	0.0000176
ti(Year,x,y)	35.6459605	96	3.7825841	0.0003680
s(ID)	20.6749079	106	0.2702253	0.0107314
s(Tmax)	22.2122466	29	18.3749948	0.0000000
s(Rain)	9.6025274	29	10.5475822	0.0000000
s(Wind)	7.4374453	29	1.8271596	0.0010261
s(Arable)	0.0006036	29	0.0000016	0.9131190
s(Urban)	1.3326238	29	0.1105030	0.0536478
s(ALAN)	0.3367692	29	0.0188473	0.1726263
s(Elevation)	0.7592636	2	1.7603392	0.0532902
s(Woodland)	0.0007024	29	0.0000033	0.8577001
s(Grassland)	0.0017996	29	0.0000470	0.3660614
ti(Tmax,Rain)	13.5260745	16	14.3336401	0.0000000
ti(Arable,ALAN)	1.2132561	16	0.2993410	0.0511700
ti(Tmax,ALAN)	7.0926743	16	4.0997758	0.1112780
ti(Woodland,Tmax)	8.7945168	16	2.1672237	0.0612280

Table: Nocturnal: 1500m

term	edf	ref.df	statistic	p.value
s(Year)	3.2912174	5	10.7409820	0.0000000
s(Year_factor)	1.2406322	7	0.6776135	0.0092300
s(Radar)	0.0000448	13	0.0000059	0.0009814
s(Year):RadarCastor-Bay	4.2606579	5	5.9342365	0.0000000
s(Year):RadarChenies	3.2162188	5	3.3304276	0.0000168
s(Year):RadarCobbacombe-Cross	0.0005231	4	0.0000461	0.4487916
s(Year):RadarCrug-y-Gorllwyn	0.0006146	4	0.0000698	0.3707608
s(Year):RadarDean-Hill	0.0003250	5	0.0000288	0.3903521
s(Year):RadarDruim-a-Starraig	0.0001557	4	0.0000438	0.0339496
s(Year):RadarHameldon-Hill	0.0011005	5	0.0001036	0.1881962
s(Year):RadarHigh-Moorsley	0.8340410	5	0.2919567	0.0355859
s(Year):RadarHill-of-Dudwick	3.9546219	5	44.4849517	0.0000000
s(Year):RadarHolehead	1.6978090	4	1.1162975	0.0006092
s(Year):RadarIngham	1.3083996	3	1.7458122	0.0016828
s(Year):RadarMunduff-Hill	2.0065328	4	2.0338079	0.0000201
s(Year):RadarPredannack	2.8640011	5	5.3534260	0.0000000
s(Year):RadarThurnham	1.6758823	5	0.8325977	0.0284846
s(Month_abb)	5.5481592	6	21.1915339	0.0000000
s(x,y)	44.0999920	99	1.9844164	0.0000000
ti(Year,x,y)	45.8615872	96	3.6119960	0.0000000
s(ID)	24.7611504	111	0.3148601	0.0058958
s(Tmax)	22.0751842	29	24.9630630	0.0000000
s(Rain)	9.8545956	29	11.5305516	0.0000000
s(Wind)	5.6308705	29	1.7172934	0.0000917
s(Arable)	0.0004928	29	0.0000052	0.6984835
s(Urban)	1.0696223	29	0.2070004	0.0605115
s(ALAN)	0.0007934	29	0.0000125	0.5168094
s(Elevation)	0.8805610	2	4.1871101	0.0520958
s(Woodland)	0.0010903	29	0.0000275	0.4295401
s(Grassland)	0.0003466	29	0.0000017	0.8814347
ti(Tmax,Rain)	13.6365410	16	22.3285391	0.0000000
ti(Arable,ALAN)	1.8210048	16	0.3334992	0.0914193
ti(Tmax,ALAN)	8.0543663	16	2.8717490	0.198776
ti(Woodland,Tmax)	11.6949381	16	4.3540472	0.0500129

Table: Nocturnal: 1700m

term	edf	ref.df	statistic	p.value
s(Year)	0.6819028	5	0.4653548	0.0045050
s(Year_factor)	2.8248209	7	2.5565070	0.0000283
s(Radar)	0.0004082	13	0.0000256	0.0942012
s(Year):RadarCastor-Bay	0.0005279	5	0.0000779	0.0663792
s(Year):RadarChenies	2.3539806	5	2.0395400	0.0014426
s(Year):RadarCobbacombe-Cross	0.0001940	4	0.0000038	0.8390493
s(Year):RadarCrug-y-Gorllwyn	0.5613312	4	0.2026974	0.0524946
s(Year):RadarDean-Hill	0.0005025	5	0.0000547	0.2948374
s(Year):RadarDruim-a-Starraig	0.5314980	4	0.2872509	0.0084090
s(Year):RadarHameldon-Hill	2.5156073	5	2.1218327	0.0000964
s(Year):RadarHigh-Moorsley	3.1978111	5	2.1869493	0.0000780
s(Year):RadarHill-of-Dudwick	4.9709180	5	104.4075876	0.0000000
s(Year):RadarHolehead	0.0003545	3	0.0000696	0.3725971
s(Year):RadarIngham	2.1090795	3	3.8362422	0.0000120
s(Year):RadarMunduff-Hill	0.0003319	4	0.0000350	0.5126801
s(Year):RadarPredannack	1.9280346	5	1.1011497	0.0064973
s(Year):RadarThurnham	1.1538523	5	0.7385908	0.0204812
s(Month_abb)	5.8554720	6	26.8754972	0.0000000
s(x,y)	12.5313811	99	0.4039290	0.0000012
ti(Year,x,y)	56.1386371	96	4.5852196	0.0000000
s(ID)	26.3608165	117	0.3356729	0.0021404
s(Tmax)	21.6174824	29	27.3505122	0.0000000
s(Rain)	7.2563538	29	4.7393166	0.0000000
s(Wind)	4.1298867	29	0.4745850	0.0821593
s(Arable)	0.4877828	29	0.0342912	0.1121761
s(Urban)	1.6430277	29	0.2138037	0.0067616
s(ALAN)	1.3528464	29	0.0938437	0.0935238
s(Elevation)	0.8715747	2	3.7974619	0.0028131
s(Woodland)	0.5055128	29	0.0369687	0.1281761
s(Grassland)	1.3543755	29	0.1161574	0.0495322
ti(Tmax,Rain)	10.6126378	16	10.4022730	0.0000000
ti(Arable,ALAN)	0.0009403	16	0.0000307	0.6226655
ti(Tmax,ALAN)	10.1214322	16	4.3790510	0.0000000
ti(Woodland,Tmax)	8.4658607	16	2.3438325	-0.0000004

Table: Nocturnal: 1900m

term	edf	ref.df	statistic	p.value
s(Year)	0.0007045	5	0.0001691	0.0624324
s(Year_factor)	1.4168602	7	0.7124645	0.0123176
s(Radar)	5.7381188	13	2.8243933	0.0000000
s(Year):RadarCastor-Bay	4.4689694	5	7.7700949	0.0000000
s(Year):RadarChenies	3.5080675	5	7.9941252	0.0000000
s(Year):RadarCobbacombe-Cross	0.0004629	4	0.0000059	0.9212011
s(Year):RadarCrug-y-Gorllwyn	2.1665212	4	3.2860658	0.0001303
s(Year):RadarDean-Hill	0.0002737	5	0.0000016	0.9803630
s(Year):RadarDruim-a-Starraig	0.0005575	4	0.0000773	0.2785021
s(Year):RadarHameldon-Hill	2.7438065	5	3.7770686	0.0000000
s(Year):RadarHigh-Moorsley	2.8999524	5	4.2100990	0.0000046
s(Year):RadarHill-of-Dudwick	4.9690755	5	96.0359576	0.0000000
s(Year):RadarHolehead	1.6455120	3	2.4852571	0.0076779
s(Year):RadarIngham	2.0618645	4	2.3931028	0.0013312
s(Year):RadarMunduff-Hill	0.2546807	4	0.0740289	0.1225567
s(Year):RadarPredannack	2.8570668	5	3.7865257	0.0000007
s(Year):RadarThurnham	0.8312305	5	1.1693700	0.0076996
s(Month_abb)	5.7762884	6	20.7251096	0.0000000
s(x,y)	0.0008829	99	0.0000068	0.4247490
ti(Year,x,y)	42.9541215	96	6.9893777	0.0000000
s(ID)	26.2248384	121	0.3040126	0.0075696
s(Tmax)	21.5712324	29	32.9638524	0.0000000
s(Rain)	11.1505343	29	6.3813956	0.0000000
s(Wind)	4.5887732	29	0.7443985	0.0143582
s(Arable)	0.4004413	29	0.0270341	0.1756282
s(Urban)	0.7947151	29	0.1740085	0.0162511
s(ALAN)	1.4650641	29	0.1091939	0.0891529
s(Elevation)	0.7385291	2	1.6670667	0.0448871
s(Woodland)	2.0229258	29	0.3633855	0.0031787
s(Grassland)	0.0003776	29	0.0000088	0.4060723
ti(Tmax,Rain)	13.9006148	16	18.4615332	0.0000000
ti(Arable,ALAN)	0.0011289	16	0.0000513	0.4527435
ti(Tmax,ALAN)	9.4875536	16	4.6083713	0.0000000
ti(Woodland,Tmax)	3.6806622	16	2.3647955	0.0000000
